# Comprehensive assessment of NR ligand polypharmacology by a multiplex reporter NR assay

**DOI:** 10.1038/s41598-022-07031-8

**Published:** 2022-02-24

**Authors:** Alexander Medvedev, Matt Moeser, Liubov Medvedeva, Elena Martsen, Alexander Granick, Lydia Raines, Kristen Gorman, Benjamin Lin, Ming Zeng, Keith A. Houck, Sergei S. Makarov

**Affiliations:** 1 Attagene Inc, Research Triangle Park, NC USA; 2 US Environmental Protection Agency, Research Triangle Park, NC USA; 3 Present Address: UNC at Chapel Hill, Chapel Hill, NC USA; 4 Present Address: Case Western Reserve University, Cleveland, OH USA

**Keywords:** Reporter genes, Hormone receptors, Steroid hormones, Toxicology, Screening, Small molecules, Receptor pharmacology

## Abstract

Nuclear receptors (NR) are ligand-modulated transcription factors that regulate multiple cell functions and thus represent excellent drug targets. However, due to a considerable NR structural homology, NR ligands often interact with multiple receptors. Here, we describe a multiplex reporter assay (the FACTORIAL NR) that enables parallel assessment of NR ligand activity across all 48 human NRs. The assay comprises one-hybrid GAL4-NR reporter modules transiently transfected into test cells. To evaluate the reporter activity, we assessed their RNA transcripts. We used a homogeneous RNA detection approach that afforded equal detection efficacy and permitted the multiplex detection in a single-well format. For validation, we examined a panel of selective NR ligands and polypharmacological agonists and antagonists of the progestin, estrogen, PPAR, ERR, and ROR receptors. The assay produced highly reproducible NR activity profiles (r > 0.96) permitting quantitative assessment of individual NR responses. The inferred EC50 values agreed with the published data. The assay showed excellent quality (<Z’>  = 0.73) and low variability (<CV> = 7.2%). Furthermore, the assay permitted distinguishing direct and non-direct NR responses to ligands. Therefore, the FACTORIAL NR enables comprehensive evaluation of NR ligand polypharmacology.

## Introduction

The human NR superfamily comprises forty-eight ligand-modulated transcription factors that regulate transcriptional responses to endocrine stimuli^1,2^. Individual NRs recognize specific hormonal and metabolic ligands and regulate different metabolic functions^1–3^. Each NR has a ligand-binding domain (LBD) that regulates the transcriptional activity and a DNA-binding domain (DBD) that interacts with the regulated genes. The NRs are readily druggable targets, and thus NR ligands comprise a large class of drugs for multiple diseases and conditions, including inflammation, contraception, diabetes, and cancer^4,5^.

A major challenge for drug development efforts stems from a considerable structural homology of NR proteins^5^. Because of that, NR drugs often interact with multiple receptors. The polypharmacological effects can compromise drug safety, but, on the other hand, they can drastically increase drug efficacy^6,7^. The proper polypharmacology evaluation requires assessing NR ligand effects on all human NRs, but tools for such comprehensive analysis are missing.

Most existing NR assays belong to two classes: the ligand-binding and the reporter gene assays^8^. The former permits evaluating ligand binding across multiple receptors but lacks information about NR activity changes. The main limitation of reporter gene assays is that they usually enable assessing only a single NR response^8–10^. The throughput can be increased using reporter cell lines with stably integrated NR reporter constructs^9,10^, but this approach is hindered by the gradual inactivation of reporter transgene expression^11^ and by unpredictable effects of surrounding chromatin.

Here, we describe a multiplex reporter assay (the FACTORIAL NR) that enables profiling NR ligands’ activity across all human NRs. The assay makes use of one-hybrid GAL4-NR reporter modules transiently transfected into test cells. The principal difference of the FACTORIAL NR from existing one-hybrid reporter assays is that we evaluate NR reporters’ activity by assessing their transcription. For that, we use a homogeneous detection approach^12^ that affords assessing multiple reporters in a single well of cells with equal detection efficacy. In our previous publications, we demonstrated the feasibility of this multiplex assay and showed its applications for screening for the endocrine disrupting activity of chemicals and water samples^13,14^. However, the prototypical (trans-FACTORIAL) assay of those studies covered only a fraction of the human NRome.

Here, we describe the comprehensive FACTORIAL NR encompassing all human NRs. To validate the assay, we assessed NR activity profiles for selective NR ligands and polypharmacological agonists and antagonists^13^. We show that these profiles permitted unequivocal identification of NR targets, and the inferred EC50 values were in agreement with the literature data. Furthermore, we have characterized the quantitative assay parameters, including the specificity, variability, quality, and repeatability. We also explored the assay's utility for dissecting direct and indirect effects of NR ligands.

## Methods

### Reagents

17β-estradiol (CAT# 10006315, CAS: 50-28-2, purity: ≥ 98%), 4-Hydroxytamoxifen (CAT# 17308, CAS: 68392-35-8, purity: ≥ 98%), Bexarotene (CAT# 11571, CAS: 153559-49-0, purity: ≥ 98%), Chenodeoxycholic acid (CAT# 10011286, CAS: 474-25-9, purity: ≥ 95%), Dexamethasone (CAT# 11015, CAS: 50-02-2, purity: ≥ 98%), Docosahexaenoic acid (DHA) (CAT# 90310, CAS: 6217-54-5, purity: ≥ 98%), DY131 (CAT# 17999, CAS: 95167-41-2, purity: ≥ 98%), Eicosapentaenoic acid (EPA) (CAT# 90110, CAS: 10417-94-4, purity: ≥ 98%), Fexaramine (CAT# 17369, CAS: 574013-66-4, purity: ≥ 98%), GSK805 (CAT# 9002444, CAS: 1426802-50-7, purity: ≥ 98%), GW0742 (CAT# 10006798, CAS: 317318-84-6, purity: ≥ 95%), GW4064 (CAT# 10006611, CAS: 278779-30-9, purity: ≥ 95%), GW590735 (CAT# 10009880, CAS: 622402-22-6, purity: ≥ 98%), GW7647 (CAT# 10008613, CAS: 265129-71-3, purity: ≥ 98%), Levonorgestrel (CAT# 10006318, CAS: 797-63-7, purity: ≥ 95%), Pioglitazone (CAT# 71745, CAS: 111025-46-8, purity: ≥ 98%), PK 11195 (CAT# 10525, CAS: 85532-75-8, purity: ≥ 98%), Progesterone (CAT# 15876, CAS: 57-83-0, purity: ≥ 98%), T0901317 (CAT# 71810, CAS: 293754-55-9, purity: ≥ 98%), Troglitazone (CAT# 71750, CAS: 97322-87-7, purity: ≥ 98%), XCT790 (CAT# 16035, CAS: 725247-18-7, purity: ≥ 98%) were purchased from Cayman Chemical (Ann Arbor, Michigan 48108 USA).

5α-Dihydrotestosterone (CAT# D-073, CAS: 521-18-6 , purity: ≥ 98%), 9-cis-Retinoic acid (CAT# R4643, CAS: 5300-03-8, purity: ≥ 98%), Aldosterone (CAT# A9477, CAS: 52-39-1, purity: ≥ 95%), Azocyclotin (CAT# 45335, CAS: 41083-11-8, purity: analytical standard), Ciglitazone (CAT# C3974, CAS: 74772-77-3 , purity: ≥ 98%), Cyhexatin (CAT# 45411, CAS: 13121-70-5, purity: analytical standard), Ethynodiol diacetate (CAT# E7263, CAS: 297-76-7, purity: ≥ 98%), Etonogestrel (CAT# SML0356, CAS: 54048-10-1, purity: ≥ 98%), Gestodene (CAT# SML0292, CAS: 60282-87-3, purity: ≥ 98%), GW501516 (CAT# SML 1491, CAS: 317318-70-0, purity: ≥ 98%), Medroxyprogesterone Acetate (CAT# PHR1589, CAS: 71-58-9, purity: analytical standard), Norgestimate (CAT# 94497, CAS: 35189-28-7, purity: analytical standard), Retinoic acid (CAT# R2625, CAS: 302-79-4, purity: ≥ 98%), Rifampicin (CAT# R3501, CAS: 13292-46-1, purity: ≥ 97%), Rosiglitazone (CAT# R2408, CAS: 122320-73-4, purity: ≥ 98%), Tributyltin chloride (CAT# T50202, CAS: 1461-22-9, purity: ≥ 96%), 3,3′,5-Triiodo-L-thyronine (CAT# T2877, CAS: 6893-02-3, purity: ≥ 95%), Triphenyltin chloride (CAT# 245712, CAS: 639-58-7, purity: ≥ 95%), 1α,25-Dihydroxyvitamin D3 (CAT# D1530, CAS: 32222-06-3, purity: ≥ 99%) were purchased from Sigma-Aldrich (St. Louis, Missouri, USA). 6α-Fluorotestosterone (cat# BML-S250-0005; CAS Number: 1597-68-8; Purity: ≥ 99.0%) was from Enzo Life Sciences (Farmingdale, NY, USA). All chemicals were dissolved in dimethyl sulfoxide (DMSO), with the final concentration of 0.2% DMSO in the cell growth media.

### Cells

As in our previous studies, the FACTORIAL NR assay was conducted in the HG19 clone (Attagene, NC, USA) of human hepatocellular carcinoma HepG2 cell line (ATCC # HB-8065). The HG19 clone had an elevated xenobiotic metabolizing activity^12–14^. Cells were propagated in a Dulbecco-modified essential culture medium (DMEM) Gibco (Waltham, MA, USA) supplemented with a 10% fetal bovine serum (FBS). The FACTORIAL NR assays were conducted in a low-serum media containing 1% charcoal-stripped FBS (Hyclone, UT, USA).

### The principle of FACTORIAL NR assay

**Figure 1:**
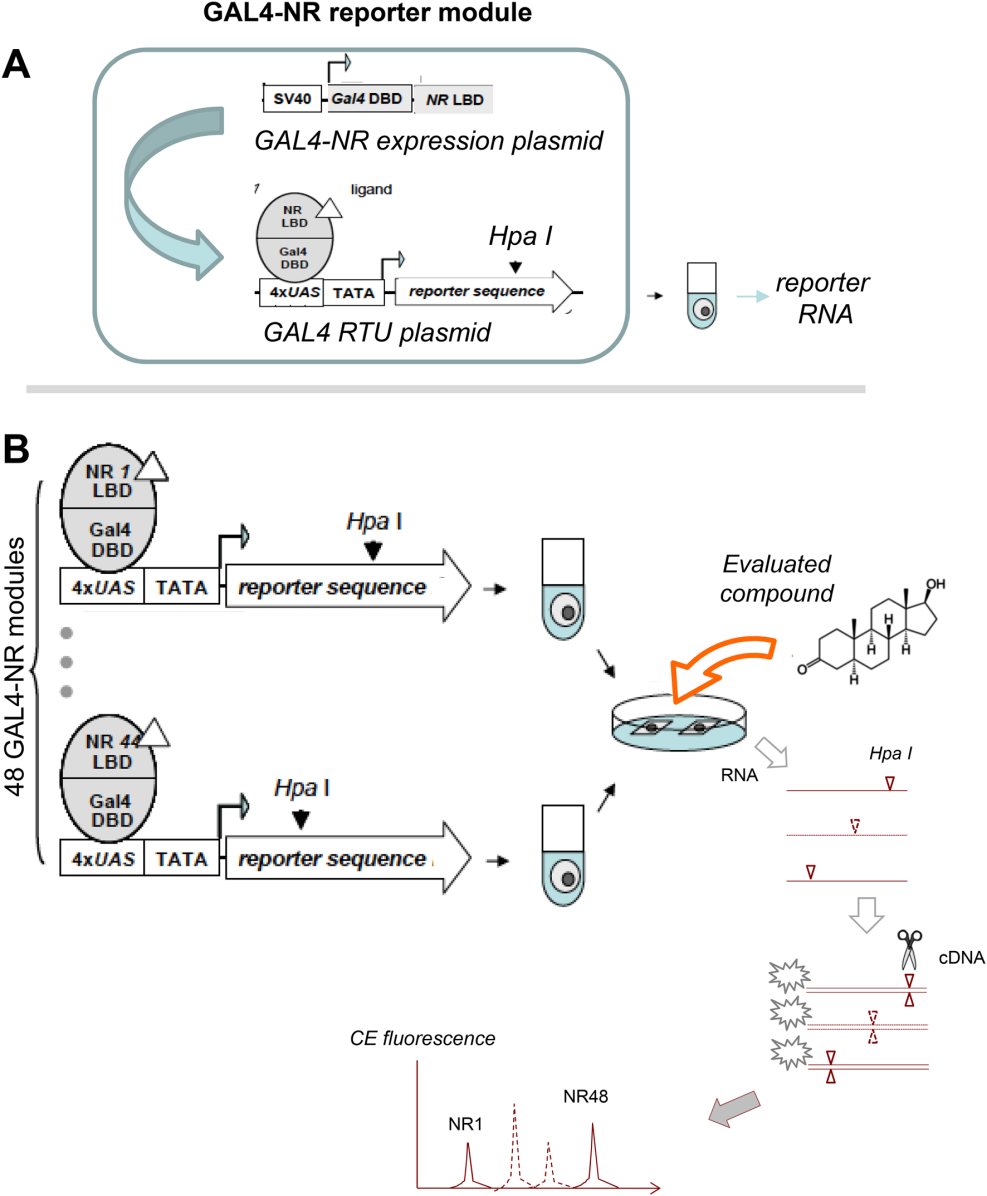
The FACTORIAL NR assay. **(A)** The GAL4-NR/RTU reporter module. The module comprises a GAL4-NR expression vector paired with a GAL4 reporter transcription unit (RTU). The module acts as a one-hybrid reporter construct producing RTU transcripts proportionate to NR LBD transcriptional activity. The RTU reporter sequence contains a restriction tag (the HpaI site). (**B)** The detection flowchart. The GAL4-NR/RTU modules are transiently transfected into separate pools of test cells. Transfected cells are mixed and plated into assay plate wells. After stimulation, total cellular RNA is amplified by RT-PCR, labeled by a fluorescent label, cut by HpaI enzyme and separated by capillary electrophoresis (CE). The CE profile mirrors the GAL4-NR activity.

The FACTORIAL NR is a modular assay comprising one-hybrid reporter modules for each human NR. An individual module has a GAL4-NR expression vector paired with a GAL4 reporter transcription unit (RTU) (Fig. 1A). The GAL4-NR vector provides constitutive expression of a chimeric GAL4-NR protein (a fusion of the NR LBD with GAL4 DBD). The RTU has a reporter sequence under the control of GAL4 DBD binding promoter. The GAL4-NR/RTU pair acts as a classic one-hybrid GAL4-NR reporter^15,16^. The GAL4-NR proteins transactivate the RTU reporter proportionate to the NR LBD activity.

To detect the FACTORIAL NR reporter modules, we profile the reporter RNA transcript using the homogeneous detection approach^12^. Under this approach, all RTUs have identical reporter sequences. To distinguish the RTU transcripts, the reporter sequences have the restriction tag (the HpaI site) placed at a different position (Fig. 1A).

The FACTORIAL NR detection entails RT-PCR amplification of reporter RNAs followed by HpaI digestion and DNA fragment separation by capillary electrophoresis (Fig. 1B). The homogeneous design ensures an equal detection efficacy across the reporters, thereby reducing the influence of experimental variables on the transcript profiles^12^.

### The GAL4-NR/RTU modules

The assay comprises 50 reporter modules. There are forty-eight reporter GAL4-NR/RTU modules, one module for each human NR. Their NR LBDs have the entire coding LBD sequences with the hinge domains. The GAL4-NR protein expression is under the control of a constitutive SV40 promoter.

To control for ligand effects on the GAL4 promoter, we used the GAL4 reporter module with the expression vector lacking an NR LBD. For internal normalization, we used the TATA module without an expression vector. Its reporter sequence was under the control of a minimal TATA box promoter.

### Validating the reporter modules

Prior to assembling the multiplex assay, we tested the functionality of individual reporter modules. All GAL4-NR expression vectors were sequence-verified. To assess the basal activity and NR ligand responsiveness, we used a traditional reporter gene assay with the secreted alkaline phosphatase (SEAP). The GAL4-NR vectors were transiently transfected along with the GAL4-SEAP reporter plasmid containing the SEAP cDNA. The SEAP activity was detected in cell growth medium following vendor’s protocol (Thermo Fisher Scientific, USA).

To test the functionality of GAL4-NR proteins with unknown NR ligands, we used two-hybrid cofactor interaction assays^17^ to determine whether the GAL4-NR proteins interact with their coactivators and corepressors to modulate reporter transcription. In these experiments, the expression plasmids contained the VP16 activation domain fused with NR coactivators (RIP140-VP16) and corepressors (NCOR-VP16 and SMRT-VP16). Note that adding the potent activating VP16 domain turns co-repressors into activators. In the case of GAL4- SHP (which has no known NR cofactors), we verified its expression by RT-PCR. The Table S1 and supplementary Fig. S1 summarize the test results.

### The assay workflow

The assay was done following the previously described protocols ^12^ (Fig. 1B). The reporter modules were transiently transfected into separate pools of HepG2 cells using the TransIT-LT1 reagent (Mirus). Twenty-four hours later, cells were trypsinized and mixed in an equal proportion. The cell mix was plated into 12-well plates at 3 × 10^5^ cells/well (each NR module being represented by approximately 6000 cells) in a 10% FBS growth medium. Each well represented an individual FACTORIAL NR assay. After the plating, cells were incubated overnight in a fresh low-serum (1% FBS) growth medium and treated with tested compounds for 24 h.

Total RNA was isolated using the PureLink RNA isolation kit (Invitrogen) and processed as described^12^. Briefly, RNA was reversely transcribed and amplified in a single PCR reaction tube using one pair of RTU primers, producing 702 bp PCR products ^12^. The products were labeled in an extension reaction with a 6-Fam-labeled primer and cut by HpaI. The minimal spacing of HpaI sites among the reporter sequences was of 5 bp to allow reliable separation of the DNA fragments by capillary electrophoresis (DNA Genetic Analyzer 3130xl (ABI)). As we showed previously, the HpaI-tagged reporters had essentially identical detection efficacy, ensuring the reproducibility of reporter transcript profiles regardless of broad variations in transfection efficacy, RNA degradation and PCR amplification^12^.

### Data analysis

The output of the FACTORIAL NR assay is the CE electrophoregram that mirrors the GAL4-NR activity. To normalize CE profiles by different assays, we used the TATA module signals. Each tested NR ligand was assessed by three independent FACTORIAL NR assays, and the NR activity profiles were calculated as an average of the three replicates. To characterize NR ligand activity, we used differential NR activity profiles calculated by dividing the NR activity values for ligand-treated cells by those in vehicle-treated cells.

### Statistical analysis

To compare NR activity profiles, we used the Pearson correlation coefficient (*r*) ^12^. The significance of individual NR responses was evaluated by Student’s t-test for the average values of three replicate assays. To assess the intraassay variability of individual NR endpoints, we used the coefficient of variation (CV) for multiple replicate assays within a single experiment using the formula below.

$${\text{CV }} = \, \left( {{\text{Standard Deviation}}/{\text{Mean induction}}} \right) \, \times { 1}00$$

As the aggregate intraassay variability <CV> , we used average CV values across all significant NR responses. To assess assay quality, we used Z'-factor values for individual NR endpoints^18^. The baseline activity in vehicle-treated cells and the activity in stimulated cells provided the negative and positive control values, respectably. As the aggregate assay quality <Z'> , we used the average of Z'-factor values across all significant NR endpoints. To calculate EC50 values, we interpolated the concentration–response curves by the curve-fitting algorithms of the 4-Parameter Logistic (4PL) model, using the DRC package (v.2.5–12)^19^ of R software (v. 3.2.5)^20^, and the SciPy package of Python (v3.62.). The NR activity profile heatmaps were generated using the Matplotlib and Seaborn modules of Python.

## Results

### The FACTORIAL NR detection parameters

#### The baseline NR activity profile

In the absence of stimulation, the activity of most GAL4-NRs was within the tenfold range of the GAL4 reporter. The RORα,β,γ, RARα,β, ERRα,γ, CAR, and SF-1 reporters had a higher activity (suppl. Fig. S2) This high constitutive activity may stem from the presence of endogenous NR ligands or from activation of signaling pathways potentiating NR activity^21^.

### The specificity

**Figure 2:**
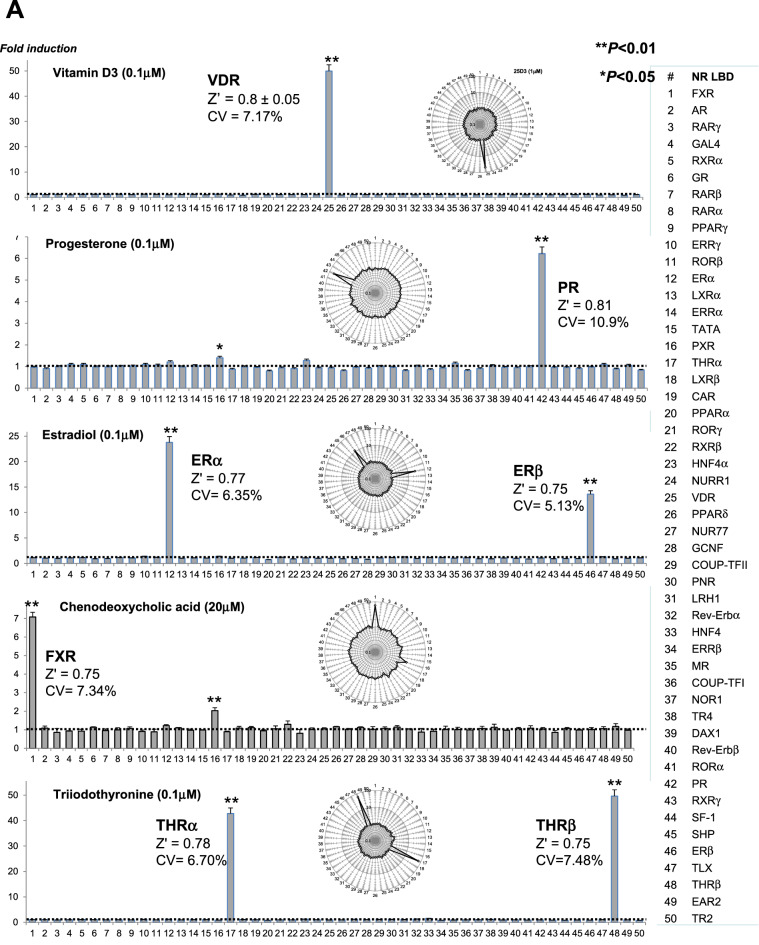

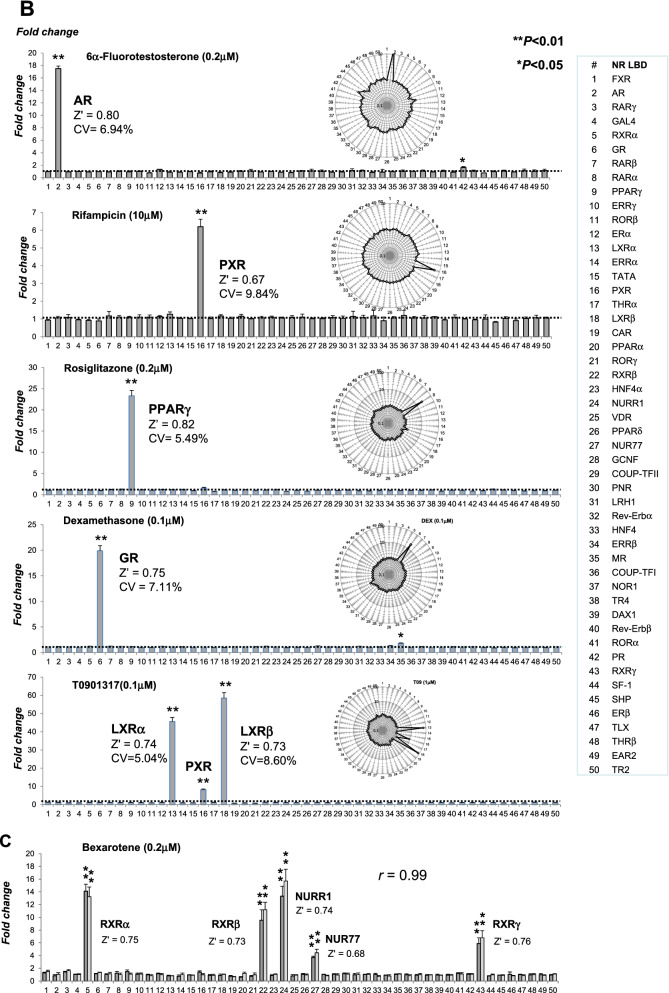
Profiling NR ligand activity by the FACTORIAL NR. The NR activity profiles for physiological **(A)** and synthetic **(B**) ligands. The NR activity profiles show the GAL4-NR activity in stimulated cells normalized by that in vehicle-treated cells. Bar graphs show NR activity fold-changes on a linear scale and radial graphs show log-transformed values. Each profile is an average of three independent FACTORIAL NR assays. Significant NR responses are marked (**P < 0.01; *P < 0.05). The Z’- factor and CV values for individual responses are averages of three independent assays. (**C)** The reproducibility of NR activity profiles for RXR agonist bexarotene in two independent experiments. Each profile is an average of three independent FACTORIAL NR assays. The profile similarity calculated as Pearson correlation coefficient *r*. The Z’-factor and CV values for individual responses are averages of three independent assays in one experiment. The aggregate < Z’ > and < CV > values are average across all significant responses.

The lack of cross-reactivity between GAL4-NR/RTU modules is a built-in feature of the assay. The reporter modules are individually transfected prior to plating the pool of transfected cells into assay wells. To further test for the specificity, we used a series of specific ligands. The panel comprised physiological NR ligands for the vitamin D (VDR), progesterone (PR), estrogen (ER), farnesoid X (FXR), and thyroid hormone (THR) receptors (Fig. 2A). The statistical significance of individual NR responses was assessed by t-test. We have found that nonspecific responses (noise) were within 3 SD from the baseline, whereas statistically significant off-target responses (e.g., PXR activation by progesterone and chenodeoxycholic acid) were in agreement with literature data^22,23^.

Figure 2B shows NR activity profiles for synthetic NR agonists of androgen (AR), pregnane X (PXR), peroxisome proliferator-activated receptor gamma (PPARγ), glucocorticoid (GR), and liver X (LXR) receptors. The profiles were entirely consistent with the reported data. For example, in addition to the primary activity at the LXRα and LXRβ, LXR agonist T0901317 had significantly activated the PXR^24^ while not affecting other reporters. Dexamethasone had significantly activated its primary target GR and the mineralocorticoid (MR) receptor^25,26^. More profiles for specific NR ligands are shown by supplementary Fig. S3. Therefore, the FACTORIAL NR assay specifically responded to the selective ligands.

### The assay variability and quality

To assess the intraassay variability, we used the CV coefficient for multiple replicate assays within a single experiment. The CV values for the prototypical ligands (as shown by Figs. 2A,B) varied from 5.04 to 10.9%, with an average value of 7.23%.

The assay quality was assessed by the Z’-factor that characterizes the separation of the induced and baseline reporter activity and the likelihood of false positives/negatives^18^. To calculate Z' values for individual endpoints, we used data of three independent replicate assays of the same experiment. The Z' factor values for Fig. 2A,B data were in the range from 0.52 to 0.82 with an average value of 0.73. That exceeds the excellent quality criterion (*Z'* > 0.5) ^18^.

### The reproducibility

For the reproducibility assessment, we compared the NR activity profile for polyvalent NR ligands from different experiments. As a quantitative measure, we used the Pearson correlation coefficient (*r*). As an example, Fig. 2C shows the signatures for a synthetic RXR agonist bexarotene^27^. The NR activity profiles were faithfully reproduced (r = 0.991) in experiments performed over several months. The signature endpoints (RXRs, Nur77, and NURR1) were in agreement with others' data^28,29^.

### Assessing NR ligand-receptor interactions

**Figure 3:**
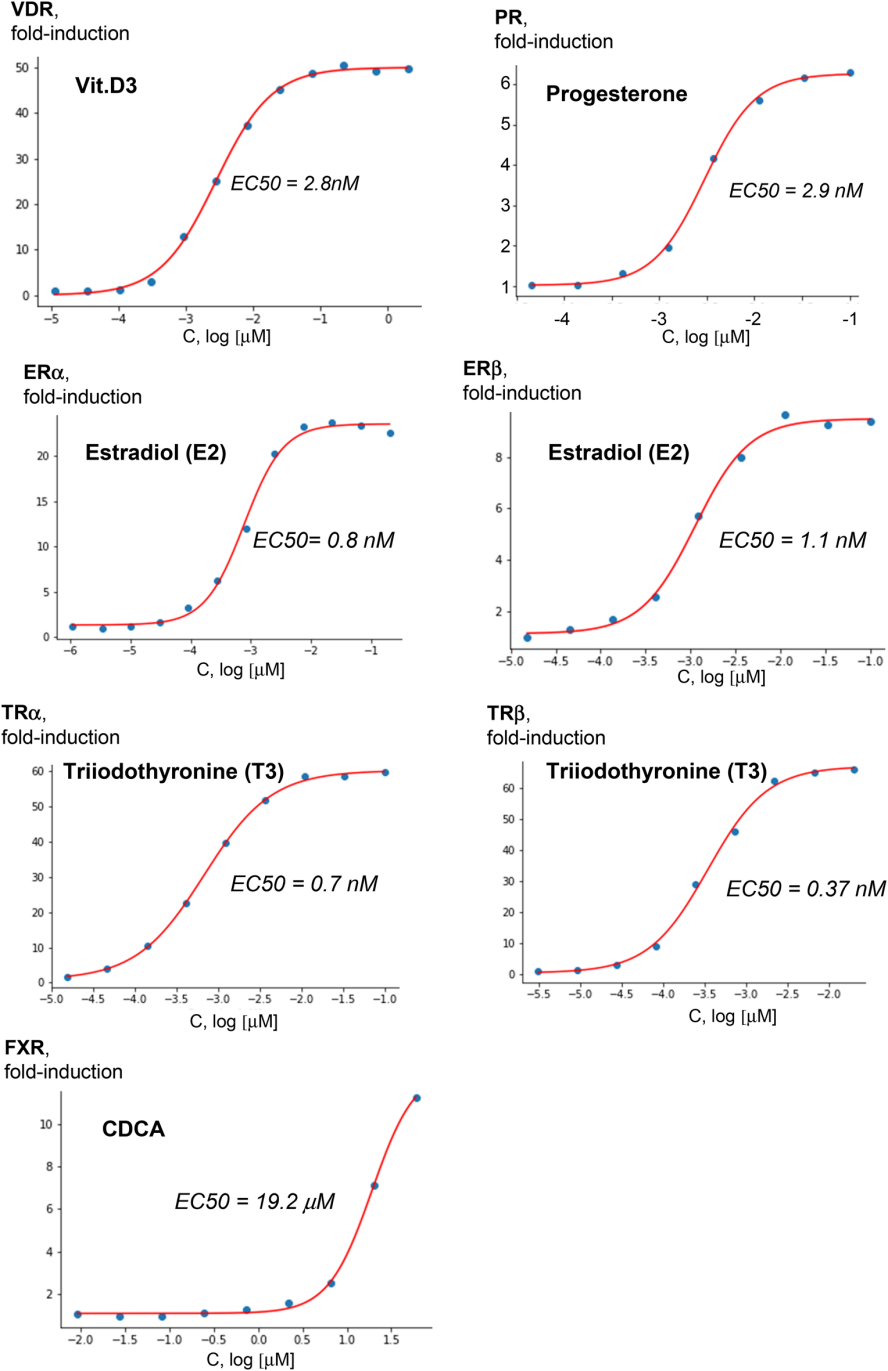

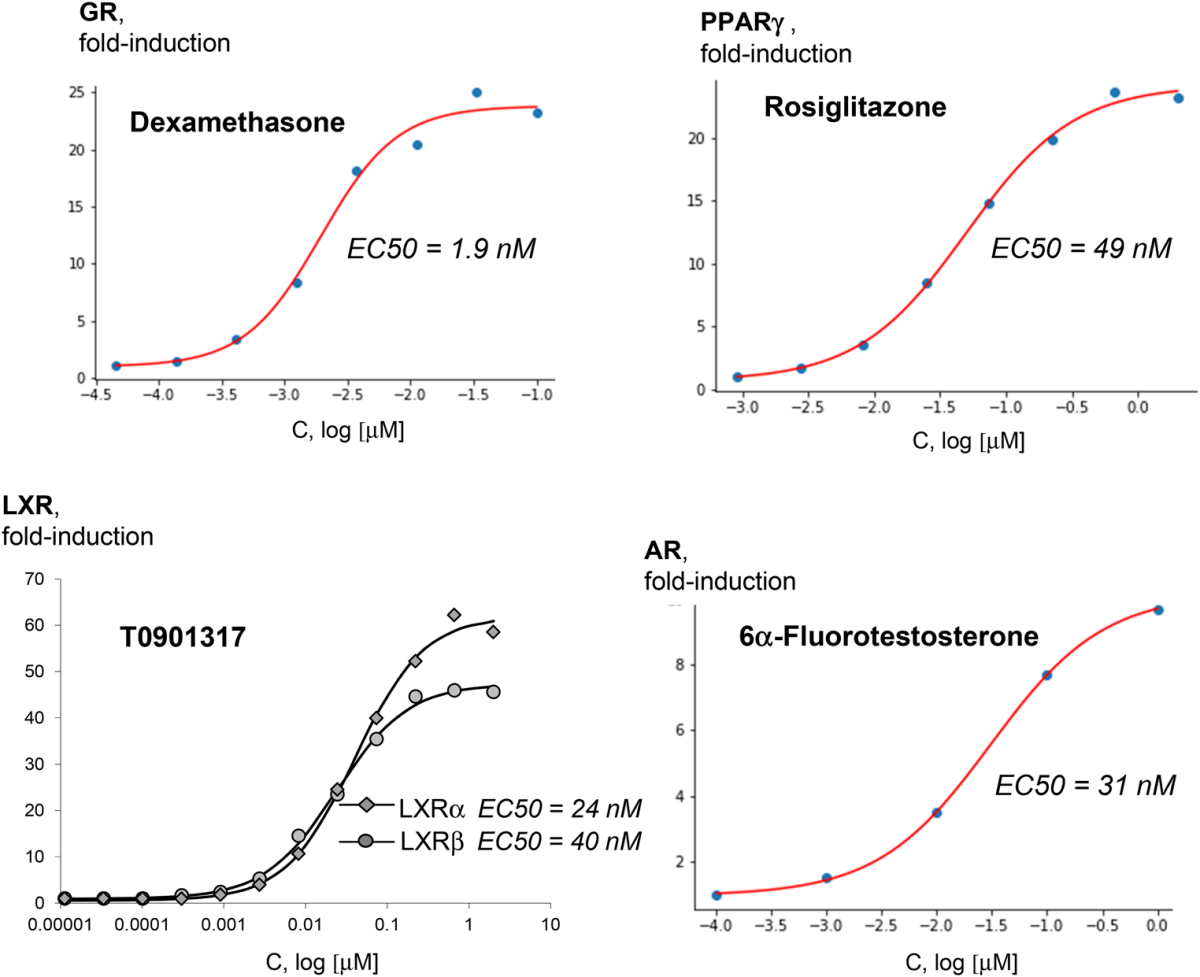
Assessing the EC50 values for ligand-receptor interactions. The concentration–response values for the ligands were interpolated by the Hill equation. The inferred EC50 values are average values of three independent FACTORIAL NR assays.

Using the FACTORIAL NR in a concentration–response format, we assessed the EC50 values for ligand-receptor interactions. The concentration–response data for selective NR ligands fitted the classic Hill equation sigmoid curves (Fig. 3). More concentration–response data are shown by supplementary Fig. S4. The inferred EC50 values agreed with the published data by others (Table 1). Therefore, the FACTORIAL NR permitted accurate quantitative evaluation of ligand-receptor interactions.

**Table 1:** The inferred EC50 values by the FACTORIAL NR assay vs. the literature data.

Compound	NR	EC50 by Factorial NR	EC50 range, literature data	References
1,25-dihydroxyvitamin D3	VDR	1.2 nM	1.0–3.28 nM	Peräkylä, 2005; Carballa, 2012
4-Hydroxytamoxifen	ERα	1.0 nM	0.5–10.3 nM	Wallace, 2003; Renaud, 2003
4-Hydroxytamoxifen	ERβ	0.8 nM	0.5–32 nM	Renaud, 2003; Wallace, 2003
4-Hydroxytamoxifen	ERRγ	22 nM	10.9–2,000 nM	Okada, 2008; Coward, 2001
Aldosterone	MR	0.24 nM	0.08–1.0 nM	Hellal-Levy, 1999; Rogerson, 1999
Bexarotene	RXRα	4.9 nM	33–40 nM	Boehm, 1994; Desphande, 2014
Bexarotene	RXRβ	2.9 nM	24 nM	Boehm, 1994
Bexarotene	RXRγ	3.1 nM	9–25 nM	Giner, 2015; Boehm, 1994
CDCA	FXR	19.2 µM	8.3–45 µM	Soisson, 2008; Houck, 2004
Dexamethasone	GR	1.9 nM	1–2.3 nM	Hellal-Levy, 1999; Rupprecht, 1993
DHT	AR	0.81 nM	0.7–8.4 nM	Zhou, 2008; Schlienger, 2009
17β estradiol	ERα	0.7 nM	0.02–3.0 nM	Schopfer, 2002; Gaido, 2000
17β estradiol	ERβ	1.1 nM	0.1–7.0 nM	Schopfer, 2002; Gaido, 2000
Fexaramine	FXR	530 nM	255 nM	Downes, 2003
GW0742	PPARα	0.9 µM	1.1–1.63 µM	Sznaidman, 2003; Nandhikonda, 2013
GW0742	PPARγ	2.8 µM	2.0–2.8 µM	Sznaidman, 2003; Nandhikonda, 2013
GW0742	PPARδ	2.2 nM	1.0–3.7 nM	Sznaidman, 2003; Nandhikonda, 2013
GW4064	FXR	88 nM	70–90 nM	Merk, 2019; Goodwin, 2000
GW7647	PPARα	3.3 nM	6.0–6.0 nM	Brown, 2001; Seimandi, 2005
GW7647	PPARγ	376 nM	350–1,100 nM	Seimandi, 2005; Brown, 2001
GW7647	PPARδ	1.26 µM	0.94–6.0 µM	Seimandi, 2005; Brown, 2001
Progesterone	PR	2.9 nM	2.2–2.9 nM	Pedram, 2008; Tegley, 1998
Rifampicin	PXR	0.73 µM	0.72–0.80 µM	Lemaire, 2006; Lehmann, 1998
Rosiglitazone	PPARγ	49 nM	18–220 nM	Seimandi, 2005; Mahindroo, 2006
T0901317	LXRα	24 nM	20–50 nM	Schultz, 2000; Li, 2017
T0901317	LXRβ	40 nM	20–60 nM	Schultz, 2000; Li, 2017
T3	THRα	0.67 nM	1.2–2.4 nM	Hofmann, 2009; Cory, 2006
T3	THRβ	0.37 nM	1.6–2.4 nM	Hofmann, 2009; Cory, 2006
XCT790	ERRα	165 nM	370–541 nM	Busch, 2004; Willy, 2004

Reference	DOI	Reference	DOI	
Boehm, 1994	10.1021/jm00044a014	Merk, 2019	10.1038/s41467-019-10853-2	
Brown, 2001	10.1016/s0960-894x(01)00188-3	Nandhikonda, 2013	10.1021/bi400321p	
Busch, 2004	10.1021/jm049334f	Okada, 2008	10.1289/ehp.10587	
Carballa, 2012	10.1021/jm3008272	Pedram, 2008	10.1021/jm8004256	
Cory, 2006	10.1021/cb600311v	Peräkylä, 2005	10.1210/me.2004-0417	
Coward, 2001	10.1073/pnas.151244398	Renaud, 2003	10.1021/jm030086h	
Desphande, 2014	10.1016/j.bmc.2013.11.039	Rogerson, 1999	10.1074/jbc.274.51.36305	
Downes, 2003	10.1016/s1097-2765(03)00104-7	Rupprecht, 1993	10.1016/0922-4106(93)90072-h	
Gaido, 2000	10.1124/mol.58.4.852	Schlienger, 2009	10.1021/jm901149c	
Giner, 2015	10.1096/fj.14-259804	Schopfer, 2002	10.1021/jm015577l	
Goodwin, 2000	10.1016/s1097-2765(00)00051-4	Schultz, 2000	10.1101/gad.850400	
Hellal-Levy, 1999	10.1016/s0014-5793(99)01667-1	Seimandi, 2005	10.1016/j.ab.2005.06.010	
Hofmann, 2009	10.1093/toxsci/kfp086	Soisson, 2008	10.1073/pnas.0710981105	
Houck, 2004	10.1016/j.ymgme.2004.07.007	Sznaidman, 2003	10.1016/s0960-894x(03)00207-5	
Lehmann, 1998	10.1172/JCI3703	Tegley, 1998	10.1021/jm980366a	
Lemaire, 2006	10.1093/toxsci/kfj173	Wallace, 2003	10.1016/s0960-894x(03)00306-8	
Li, 2017	10.1124/mol.116.105213	Willy, 2004	10.1073/pnas.0401420101	
Mahindroo, 2006	10.1021/jm0510373	Zhou, 2008	10.1038/bjp.2008.107	

The table shows the inferred EC50 values by the FACTORIAL NR assay vs. the literature data. The EC50 estimates by the FACTORIAL NR are average values of at least three independent replicate assays.

### Examining NR antagonists

**Figure 4:**
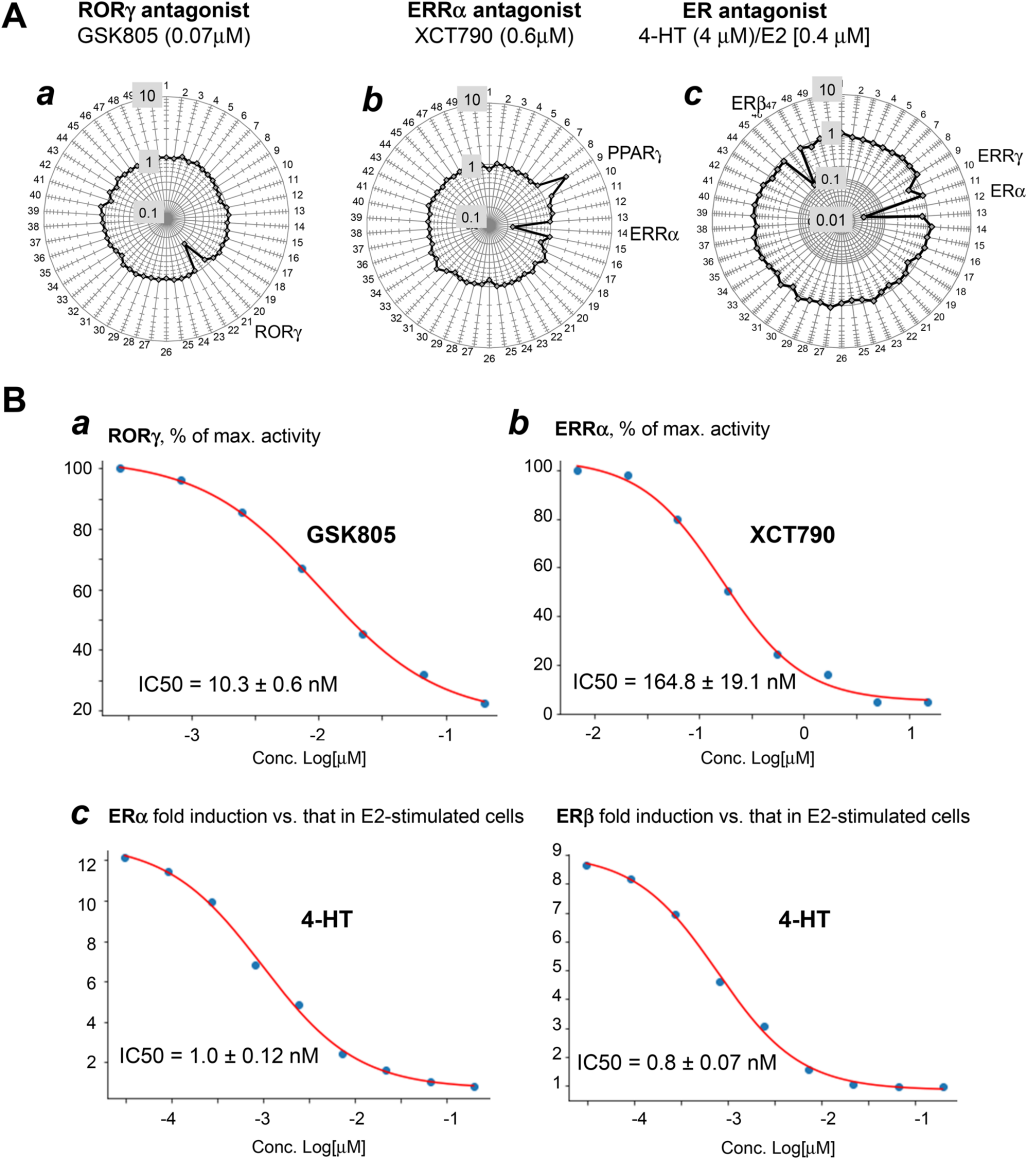
Assessing NR antagonists by the FACTORIAL NR assay. **(A)** The NR activity profiles for RORγ (**a**), ERRα (**b**), and ER (**c**) antagonists after a 24-h incubation. The log-transformed fold-changes of NR activity in antagonist- vs. vehicle-treated cells are shown. (**c**) To assess ER antagonist 4-HT, cells were stimulated with ER agonist 17β-estradiol (E2). The differential NR activity profile (**c**) shows NR activity changes in cells treated with the combination of E2/4-HT vs. that in E2-treated cells. The (**a–c)** profiles are average of three independent replicate FACTORIAL NR assays. (**B**) The concentration-responses of the primary NR targets of RORγ (**a**), ERRα (**b**), and ER (**c**) antagonists in FACTORIAL NR assay. The responses show the percentage of the baseline activity in vehicle-treated (**a,b**). or (**c**) E2-stimulated cells. The inferred EC50 values are average data of three independent FACTORIAL NR assays.

The rapid turnover of reporter RNA makes the FACTORIAL NR assay particularly well-suited for detecting inhibited NR responses to NR antagonists. Figure 4A shows the NR activity profiles for ERRα, RORγ, and ER antagonists. The high basal activity of ERRα and RORγ reporters (suppl. Fig. S2) allowed assessing their antagonists without additional stimulation. The NR activity profiles agreed with the literature data on these antagonists. The single NR response to RORγ antagonist GSK805 reflected its primary activity^30^ (Fig. 4Aa). The ERRα antagonist XCT79027 had inhibited the primary target (ERRα) and activated the PPARγ (Fig. 4Ab). The PPARγ response may be explained by XCT790 effects on PPARγ coactivator 1-alpha (PGC-1α)^31^.

The ER antagonist 4-hydroxytamoxifen (4-HT) is the major active metabolite of tamoxifen^32^. Since ER reporters had low basal activity (suppl. Fig. S3), we stimulated cells with ER agonist 17β estradiol (E2). The NR activity profile for 4-HT showed an inhibition of ERα and ERβ and ERRγ (Fig. 4Ac). The ERRγ response to 4-HT was consistent with the reported data^33^.

Using the assay in a concentration–response format, we determine EC50 values for the NR antagonists (Fig. 4Ba–c). The inferred values were in agreement with the literature data (Table 1).

These results demonstrate excellent quality (Z’ factor), low intraassay variability (CV), high reproducibility (r), and the robustness of the FACTORIAL NR assay. Furthermore, these data show the assay’s capability for evaluating both NR agonists and antagonists.

### Examining polypharmacological NR ligands

We used a diverse panel of polypharmacological PR and PPAR ligands, including drugs, nutritionals, and environmental chemicals.

### PR agonists

Progestins are synthetic analogs of the hormone progesterone, widely used in contraception pills and hormone replacement therapy^34^. The primary progestin target is the PR receptor, but they have activities at other NRs.

**Figure 5:**
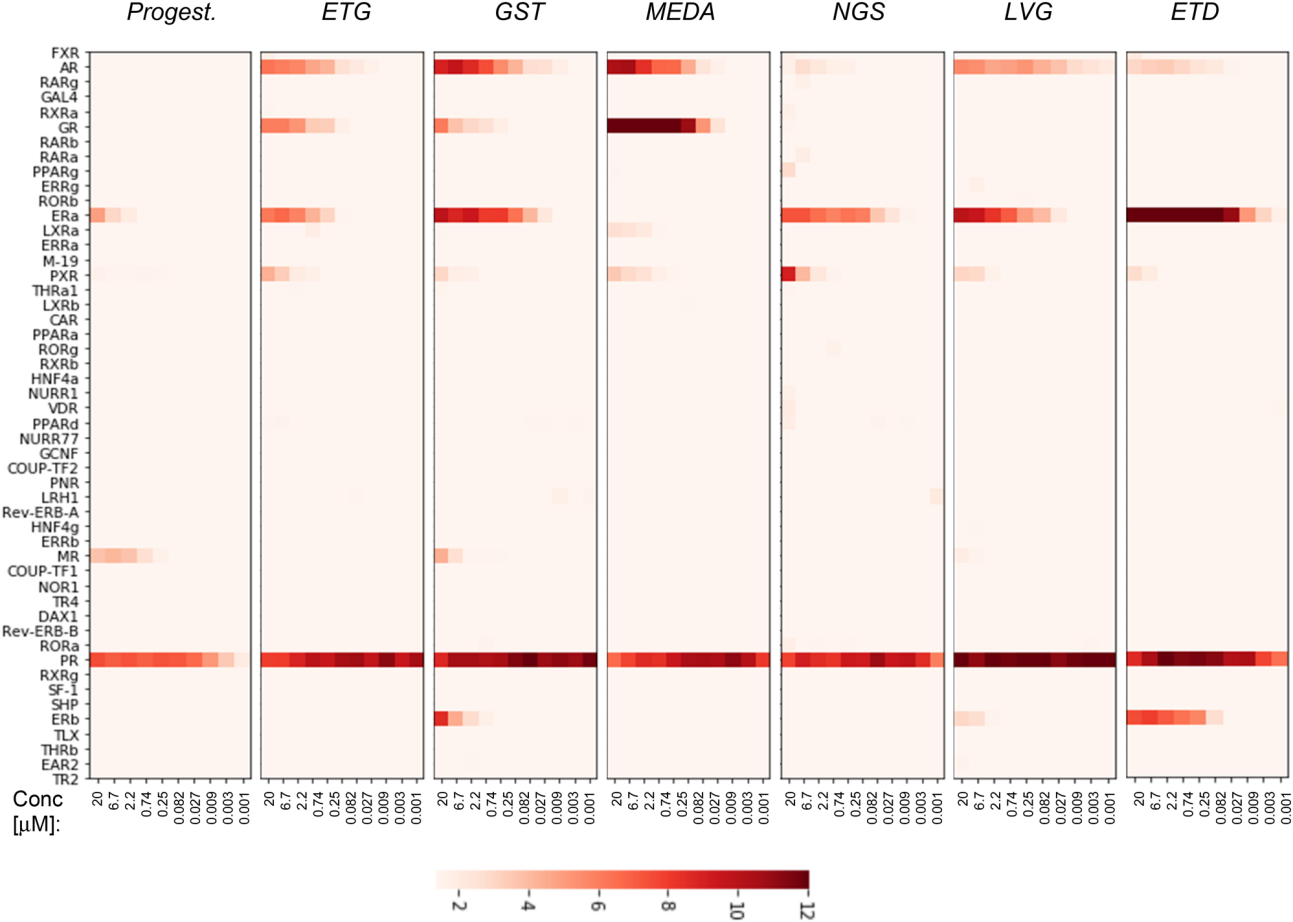
Assessing the polypharmacology of progestins. The heatmap shows NR activity profiles for progestins after a 24 h incubation with indicated concentrations. The fold-induction NR activity values in progestins vs. vehicle-treated cells are shown. *Progest.* progesterone; *ETG* etonogestrel, *GST* gestodene; *MEDA* medroxyprogesterone acetate; *NGS* norgestimate; *LVG* levonorgestrel; *ETD* ethynodiol diacetate; *PRG* progesterone.

**Figure 6:**
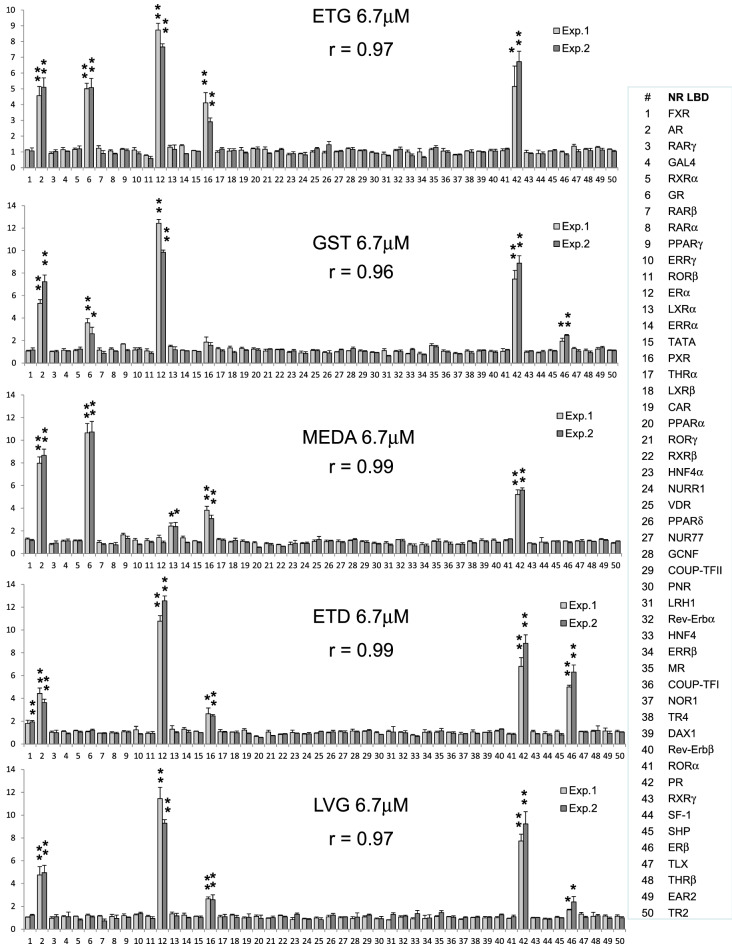
The reproducibility of progestins’ NR activity profiles. The NR data of two independent experiments are shown. Each profile is an average of three independent replicate FACTORIAL NR assays. All significant individual NR responses are marked by asteriscs (**P < 0.01; *P < 0.05). The similarity of NR activity profiles is calculated as the Pearson correlation coefficient *r*.

Here, we evaluated NR activity profiles for a progestin panel that included etonogestrel (ETG), gestodene (GST), medroxyprogesterone acetate (MEDA), norgestimate (NGS), levonorgestrel (LVG), ethynodiol diacetate (ETD). The Fig. 5 heatmap exemplifies progestins’ NR activity profiles (for more data see suppl. Fig. S5A–C). These profiles were faithfully reproduced (r > 0.96) in experiments conducted over the period of several months (Fig. 6).

The most common off-target responses involved the AR, ER, GR, and PXR receptors, which agreed with the literature data^35–38^ (Fig. 5). These effects varied among progestins, e.g., NGS had a weak activity at the AR and GR; MEDA showed no estrogenic activity; ETD activated the FXR receptor (Fig. 5 and suppl. Fig. S5A–C).

**Figure 7:**
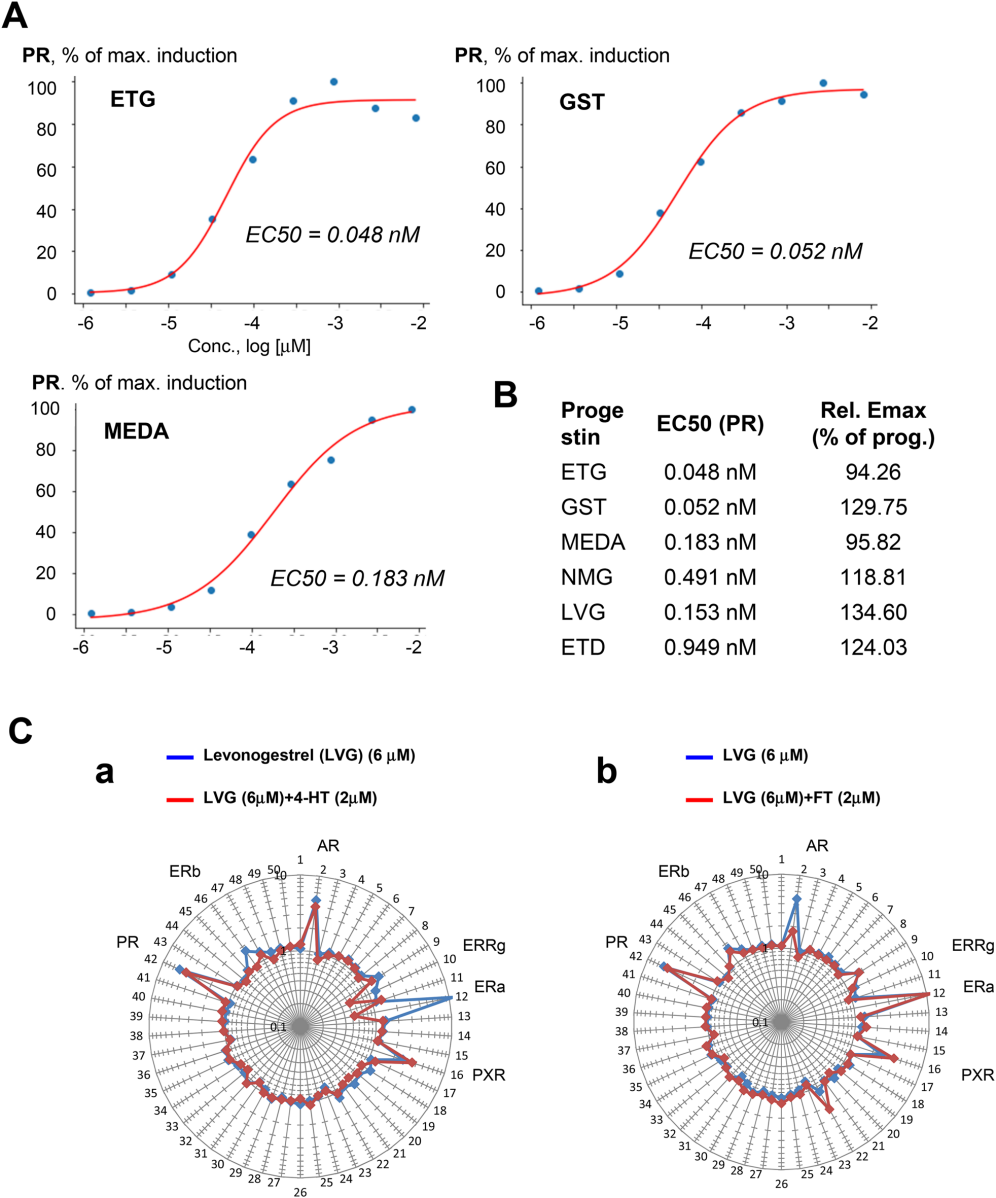
Evaluating the NR activity of progestins. **(A)** The concentration–response of progestins’ primary target (the PR). The data shown as the percentage of the maximal PR activation by the progestins. Average data of three independent replicate FACTORIAL NR assays are shown. (**B)** The inferred EC50 values for the primary progestin activity. (**C)** A competitive mode assay to assess off-target activity mechanisms. The graphs show log-transformed NR activity fold-changes (in progestin- vs. vehicle-treated cells) after a 24-h treatment with LVG. Blue line shows the NR activity profile for LVG. Red line*:* the NR activity profile for LVG in the presence of ER inhibitor 4-HT (**a**) or AR inhibitor FT (**b**). Each is the average profile of three independent replicate FACTORIAL NR assays.

The inferred on-target activity EC50 values at the PR varied among progestins from 0.048 to 0.95 nM (Fig. 7A,B). The potency of synthetic progestins was similar or higher as compared to that of progesterone (Fig. 7B, S5B).

Our data illustrate the diversity of endocrine disrupting properties of synthetic progestins. The polypharmacology may account for the differential clinical side effects^35,36^. These data also demonstrate the utility of the FACTORIAL NR for selecting optimal drug candidates with appropriate activity profiles.

To examine the underlying mechanisms of the off-target effects, we used the assay in a competitive mode. An example of this approach is the study of levonorgestrel (LVG). The off-target LVG activity was at the PXR, AR, ERα, and ERβ receptors (Fig. 5). These responses can stem from direct effects of ligand binding or from activation of cellular pathways that potentiate the NR activity. To distinguish between these possibilities, we assessed these responses in the presence of NR antagonists. The ER antagonist 4-HT had inhibited ERα and ERβ activation, but not other NR responses (Fig. 7Ca). (For other progestins’ data see suppl. Fig. S7). Akin to that, AR antagonist flutamide selectively inhibited LVG effects at the AR (Fig. 7Cb). Therefore, ER and AR activation were direct effects of LVG receptor binding.

### PPAR agonists

**Figure 8:**
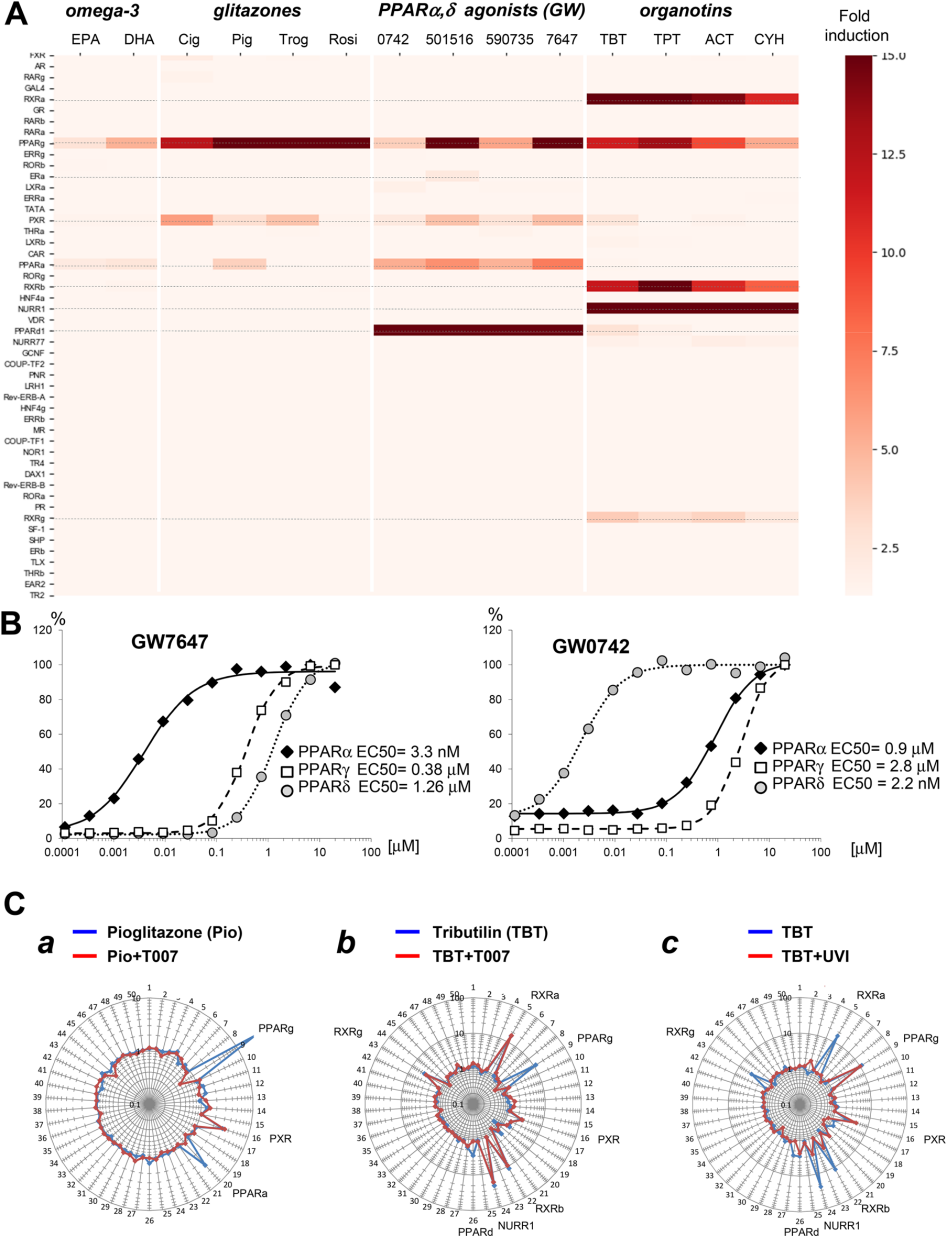
Assessing the polypharmacology of PPAR agonists. **(A)** NR activity profiles after a 24 h incubation with indicated concentrations of PPAR ligands. The heatmap shows fold-induction NR activity values in stimulated vs. vehicle-treated cells. Each profile is an average of three independent replicate FACTORIAL NR assays. *EPA* eicosapentaenoic acid; *DHA* docosahexaenoic acid; *Cig* ciglitazone; *Pio* pioglitazone; *Trog* troglitazone; *Rosi* rosiglitazone; *TBT* tributyltin; *TPT* triphenyltin; *ACT* azocyclotin; *CYH* cyhexatin. Organotins were used at 0.1 µM as they were cytotoxic at higher concentrations; all other inducers were at 5 µM. (**B)** Concentration–response of PPAR isoforms to PPARα agonist GW7647 and PPARδ agonist GW0742. Average data of three independent replicate FACTORIAL NR assays. (**C)** Examining off-target activity mechanisms for PPAR ligands. The blue line graphs show NR activity fold-changes in response to Pio (at 5 µM) (**a**) or TBT (0.1 µM) (**b**), (**c**) vs. vehicle-treated cells. The red line graphs show NR activity profiles for the PPAR ligands in the presence of PPAR inhibitor T0070907 (2 µM) (**a**), (**b**) or RXR inhibitor UVI3003 (2 µM) (**c**) vs. vehicle-treated cells. Average profiles of three independent replicate FACTORIAL NR assays are shown.

We have evaluated four classes of PPAR agonists, including nutritional ligands (omega-3 fatty acids); pharmacological PPARγ agonists (glitazones); selective agonists of PPARα and PPARδ; and environmental PPAR ligands (organotins). To capture the maximal range of off-target activities, we used high concentrations of these ligands (Fig. 8A).

The PPAR ligands produced four distinct signature clusters. The first cluster comprised signatures of most common omega-3 acids, the eicosapentaenoic acid (EPA) and docosahexaenoic acid (DHA)^39^. Their NR activity profiles were characterized by PPARγ and PPARα responses, which was consistent with the literature data^40^.

The second cluster comprised antidiabetic drugs pioglitazone, ciglitazone, troglitazone, and rosiglitazone. All the glitazones had induced PPARγ activation, reflecting their primary activity^41^. Another common response to glitazones, the PXR activation, was also in agreement with the literature data^42^. Interestingly, one drug (pioglitazone) had activated the PPARα; this response was further confirmed by competitive experiments (see Fig. 8Ca).

The third cluster comprised selective agonists of PPARα (GW59073540 and GW764741) and PPARδ (GW074242 and GW50151643). At low concentrations, GW7647 and GW0742 selectively activated PPARα and PPARδ, respectively (Fig. 8B), which was consistent with their selectivity ^43,44^. At a high (5 µM) concentration, all these compounds activated PPARα, δ, and γ, and PXR. In addition, GW501516 showed a weak estrogenic activity. The concentration-responses curves fitted the classic Hill’s equation (Figs. 8B and S8A). The inferred EC50 values for these compounds agreed with the literature data (Table 1).

The fourth signature cluster comprised widely used industrial chemicals organotins^45^. The consensus signature comprised responses of multiple NRs, including PPARγ, NURR1, and RXRα, β, and γ (Figs. 8A and S6). These organotin-induced responses were in agreement with the literature data^45,46^. We have also detected some specific features, e.g., PPARδ and PXR activation by tributyltin chloride. Our data support the notion that the endocrine disrupting activity significantly contributes to organotins’ toxicity^45,46^. These data also illustrate the utility of FACTORIAL NR for assessing polypharmacological environmental contaminants.

To examine the underlying mechanisms for the NR responses to the PPAR ligands, we used the assay in a competitive mode (Figs. 8C and S8B). To this end, we used a selective PPARγ antagonist T0070907^47^ that acts as a pan-PPAR inhibitor at higher concentrations. The addition of T0070907 had inhibited the PPARγ and PPARα responses to pioglitazone (Fig. 8Ca) and PPARδ activation by tributyltin (TBT) (Fig. 8Cb). Therefore, these responses stemmed from direct binding of these compounds to the PPAR LBDs.

To assess TBT-induced responses, we used a selective RXR antagonist UVI3003^48^. Its addition had inhibited the activation of both RXRs and NURR1 (Fig. 8Cc). Therefore, RXR activation by TBT was mediated by a direct LBD binding, whereas the NURR1 response had involved an RXR-dependent mechanism, presumably the NURR1-RXR dimerization^49^.

Summarily, these results demonstrate the utility of FACTORIAL NR assay for the evaluation of polypharmacological NR ligands. Specifically, we have shown that the assay afforded clear-cut identification of their multiple NR targets and permitted accurate quantitative assessments of the EC50 values. Moreover, the assay provided valuable mechanistic insights into ligands’ activity.

## Discussion

Currently, reporter gene assays are the gold standard for NR ligand evaluation^8^. However, most of these assays evaluate only a single NR response. Because of that, screening NR ligands across multiple receptors necessitates large assay panels. That requires protracted assay development, time, and expense. Moreover, these screens may not always be appropriately controlled, complicating the analysis across experimental variables^50,51^.

The FACTORIAL NR obviates the main limitation of traditional low-content reporter gene assays. Using the transcription-based detection enabled parallel assessment of multiple reporters in a single-well format. In addition, the homogeneous design has equalized the detection efficacy across the reporters, thereby drastically diminishing the influence of variable experimental conditions^12^. Another essential feature is the use of transient transfection to eliminate the unpredictable effects of the host genome on the reporters.

The inherent robustness has translated into excellent assay quality (< Z’ >  = 0.73 > and low intraassay variability (< CV >  = 7.2%). Furthermore, the obtained NR activity profiles were faithfully reproduced (r > 0.96) in experiments conducted over a several-month period. Thus, these signatures afforded unequivocal identification of NR targets for polypharmacological ligands. Furthermore, the FACTORIAL NR enabled quantitative assessments of ligand-receptor interactions. What is important, the inferred EC50 values agreed with the literature data. However, it should be noted that the EC50 estimates by different groups often differed by orders of magnitude (Table 1). That may be explained by differences in the reporter assays and experimental conditions. In this regard, the advantage of the FACTORIAL NR assay is that it permits parallel evaluation of EC50 values for multiple ligand-receptor interactions, thereby minimizing the interassay variability.

Another advantage of the FACTORIAL NR over the traditional reporter gene assays is rapid turnover of reporter RNAs. That results in faster responsiveness, which is particularly useful for detecting NR inhibition and evaluating NR antagonists.

Our previous publications have shown the ramifications of a prototype multiplex NR assay (trans-FACTORIAL) for assessing environmental chemicals^14^, water pollution^13^, and NR drugs^52^. However, the prototype assay covered only a fraction of human NRs. In this regard, the FACTORIAL NR enables profiling NR ligands across the entire human NRome, thus permitting comprehensive environmental and pharmacological NR ligands evaluation. Notably, the FACTORIAL NR provides valuable mechanistic insights into NR ligand activity. As we showed, using the assay in a competitive mode permitted distinguishing direct and indirect effects of polypharmacological ligands on multiple NR targets (Figs. 7C, 8C, suppl. Figs. S7, S8B).

Like any assay, the FACTORIAL NR has its limitations. The advantage of using ectopically expressed GAL4-NR proteins is that they permit assessing NR ligands’ activity across the entire NRome. On the other hand, this approach does not capture the complete spectrum of compounds’ effects on the endogenous receptors, e.g., on NR transcription, post-transcriptional modifications, splice variants, receptor biosynthesis, and metabolism. Also, the GAL4-NR proteins may not detect the allosteric NR modulators binding the NR protein outside of the LBD region.

To address these deficiencies, we use a complementary multiplex reporter assay, the cis-FACTORIAL (a.k.a. FACTORIAL TF). Unlike the FACTORIAL NR, the cis-FACTORIAL assay comprises a set of reporters that enable profiling the activity of endogenous TF families^12,14,53^. However, the cis-FACTORIAL does not allow distinguishing the individual NR responses (i.e., of α, β, γ, δ receptors). Besides, the cis-assay’s content is limited by the NRs expressed by a particular cell type. Therefore, combining the FACTORIAL NR and cis-FACTORI AL assays provides the most comprehensive evaluation of compounds’ acti vity.

## Supplementary Information

Supplementary Information.

## Data Availability

The authors declare that the all data needed to support the conclusions of this study are available in the paper and the supplementary information. The raw data are available from the corresponding author upon reasonable request.
