# Supplementary material for: Comprehensive assessment of NR ligand polypharmacology by a multiplex reporter NR assay
Source: Sci Rep. 2022 Feb 24;12:3115. doi: 10.1038/s41598-022-07031-8 (PMC8873415; doi:10.1038/s41598-022-07031-8)
Supplement: Supplementary file 1 — Supplementary Information. [file 41598_2022_7031_MOESM1_ESM.pdf]

SUPPLEMENTARY DATA

Comprehensive assessment of NR ligand polypharmacology by a multiplex reporter NR assay.

Alexander Medvedev[1], Matt Moeser[1,2], Liubov Medvedeva[1], Elena Martsen[1], Alexander Granick[1], Lydia Raines[1,3], Kristen Gorman[1], Benjamin Lin[1], Ming Zeng[1], Keith A. Houck[4], Sergei S. Makarov[1,*]

[1]Attagene Inc, Research Triangle Park, North Carolina, USA
[2]Current address: UNC at Chapel Hill, Chapel Hill, North Carolina, USA
[3]Current address: Case Western Reserve University, Cleveland, OH, USA
[4]US Environmental Protection Agency, Research Triangle Park, North Carolina, USA

*Corresponding author: Sergei S. Makarov, Ph.D.
Attagene Inc, Research Triangle Park, North Carolina, USA
smak@attagene.com (SSM)

#	NR	Nomencl.	Inducer	GAL4-NR Activity (fold)	Assay
1	**AR**	NR3C4	GAL4-AR	0.87 ± 0.04	one-hybrid assay
			6a-Fluorotestosterone	77.45 ± 11.4	agonist
2	**CAR**	NR1I3	GAL4-CAR	193.07 ± 7.6	one-hybrid assay
			PK 11195	0.11 ± 0.01	antagonist
3	**COUP-TFI**	NR2F1	GAL4-COUP-TFI	0.41 ± 0.01	one-hybrid assay
			RIP140-VP16	109 ± 3	two hybrid assay
4	**COUP-TFII**	NR2F2	GAL4-COUP-TFII	0.39 ± 0.01	one-hybrid assay
			RIP140-VP16	58.3 ± 2.0	two hybrid assay
5	**DAX-1**	NR0B1	GAL4-DAX-1	0.3 ± 0.01	one-hybrid assay
			RIP140-VP16	8.6 ± 0.2	two hybrid assay
6	**EAR2**	NR2F6	GAL4-EAR2	0.3 ± 0.02	one-hybrid assay
			RIP140-VP16	161 ± 9	two hybrid assay
7	**ERRα**	NR3B1	GAL4-ERRa	5.02 ± 0.27	one-hybrid assay
			XCT790	0.15 ± 0.01	antagonist
8	**ERRβ**	NR3B2	GAL4-ERRb	21.55 ± 0.36	one-hybrid assay
			DY131	2.6 ± 0.1	agonist
9	**ERRγ**	NR3B3	GAL4-ERRg	33.21 ± 0.61	one-hybrid assay
			4-Hydroxytamoxifen	0.21 ± 0.01	antagonist
10	**ERα**	NR3A1	GAL4-ERa	0.47 ± 0.03	one-hybrid assay
			Estradiol	142.18 ± 14.66	agonist
			4-Hydroxytamoxifen	0.21 ± 0.01	antagonist
11	**ERβ**	NR3A2	GAL4-ERb	0.51 ± 0.02	one-hybrid assay
			Estradiol	49.61 ± 0.79	agonist
12	**FXR**	NR1H4	GAL4-FXR	0.8 ± 0.05	one-hybrid assay
			CDCA	176.51 ± 9.2	agonist
13	**GCNF**	NR6A1	GAL4-GCNF	0.21 ± 0.01	one-hybrid assay
			SMRT-VP16	16.9 ± 1.8	two hybrid assay
14	**GR**	NR3C1	GAL4-GR	1.57 ± 0.05	one-hybrid assay
			Dexamethasone	67.58 ± 0.55	agonist
15	**HNF4α**	NR2A1	GAL4-HNF4a	14.1 ± 0.86	one-hybrid assay
			RIP140-VP16	31.2 ± 3.6	two hybrid assay
16	**HNF4γ**	NR2A2	GAL4-HNF4g	6.8 ± 0.49	one-hybrid assay
			RIP140-VP16	18.1 ± 2.1	two hybrid assay
17	**LRH-1**	NR5A2	GAL4-LRH-1	3.45 ± 0.59	one-hybrid assay
			RIP140-VP16	43.9 ± 8.1	two hybrid assay
18	**LXRα**	NR1H3	GAL4-LXRa	2.38 ± 0.1	one-hybrid assay
			T0901317	112.32 ± 2.78	agonist
19	**LXRβ**	NR1H2	GAL4-LXRb	0.92 ± 0.06	one-hybrid assay
			T0901317	313.93 ± 7.09	agonist
20	**MR**	NR3C2	GAL4-MR	1.44 ± 0.04	one-hybrid assay
			Aldosterone	12.27 ± 0.36	agonist
21	**NOR1**	NR4A3	GAL4-NOR1	36.92 ± 0.69	one-hybrid assay
			RIP140-VP16	1.93 ± 0.08	two hybrid assay
22	**NUR77**	NR4A1	GAL4-NUR77	1.6 ± 0.02	one-hybrid assay
			9-cis-Retinoic acid	9.29 ± 0.11	agonist
23	**NURR1**	NR4A2	GAL4-NURR1	105.64 ± 4.41	one-hybrid assay
			9-cis-Retinoic acid	54.97 ± 0.75	agonist
24	**PNR**	NR2E3	GAL4-PNR	0.34 ± 0.01	one-hybrid assay
			RIP140-VP16	3.1 ± 0.2	two hybrid assay

Table S1

25	**PPARα**	NR1C1	GAL4-PPARa	6.02 ± 0.44	one-hybrid assay
			GW-7647	21.11 ± 1.2	agonist
26	**PPARγ**	NR1C3	GAL4-PPARg	5.29 ± 0.19	one-hybrid assay
			Rosiglitazone	43.49 ± 0.76	agonist
27	**PPARδ**	NR1C2	GAL4-PPARd	0.2 ± 0.01	one-hybrid assay
			GW-0742	37.2 ± 0.73	agonist
28	**PR**	NR3C3	GAL4-PR	1.04 ± 0.04	one-hybrid assay
			Progesterone	14.78 ± 0.72	agonist
29	**PXR**	NR1I2	GAL4-PXR	0.33 ± 0.01	one-hybrid assay
			Rifampicin	43.9 ± 1.74	agonist
30	**RARα**	NR1B1	GAL4-RARa	42.05 ± 0.19	one-hybrid assay
			Retinoic acid	9.02 ± 0.19	agonist
31	**RARβ**	NR1B2	GAL4-RARb	45.24 ± 1.21	one-hybrid assay
			Retinoic acid	4.92 ± 0.04	agonist
32	**RARγ**	NR1B3	GAL4-RARg	6.77 ± 0.3	one-hybrid assay
			Retinoic acid	12.61 ± 0.49	agonist
33	**Rev-erbα**	NR1D1	GAL4-Rev-erba	0.67 ± 0.01	one-hybrid assay
			SMRT-VP16	703 ± 85	two hybrid assay
34	**Rev-erbβ**	NR1D2	GAL4-Rev-erbb	0.7 ± 0.02	one-hybrid assay
			NCOR-VP16	65 ± 6	two hybrid assay
35	**RORα**	NR1F1	GAL4-RORa	79.03 ± 6.77	one-hybrid assay
			RIP140-VP16	1.11 ± 0.12	two hybrid assay
36	**RORβ**	NR1F2	GAL4-RORb	375.5 ± 1.33	one-hybrid assay
			RIP140-VP16	1.37 ± 0.17	two hybrid assay
37	**RORγ**	NR1F3	GAL4-RORg	238.07 ± 10.34	one-hybrid assay
			GSK805	0.22 ± 0.01	antagonist
38	**RXRα**	NR2B1	GAL4-RXRa	0.54 ± 0.02	one-hybrid assay
			9-cis-Retinoic acid	32.22 ± 1.27	agonist
39	**RXRβ**	NR2B2	GAL4-RXRb	0.89 ± 0.02	one-hybrid assay
			9-cis-Retinoic acid	28.43 ± 2.82	agonist
40	**RXRγ**	NR2B3	GAL4-RXRg	0.79 ± 0.02	one-hybrid assay
			9-cis-Retinoic acid	21.84 ± 1.33	agonist
41	**SF1**	NR5A1	GAL4-SF1	98.49 ± 9.45	one-hybrid assay
			RIP140-VP16	2.5 ± 0.05	two hybrid assay
42	**SHP**	NR0B2	GAL4-SHP	0.31 ± 0.01	one-hybrid assay
			N/A	n/a	RNA expression
43	**TLX**	NR2E2	GAL4-TLX	0.23 ± 0.01	one-hybrid assay
			RIP140-VP16	8.9 ± 0.7	two hybrid assay
44	**TR2**	NR2C1	GAL4-TR2	1.19 ± 0.02	one-hybrid assay
			RIP140-VP16	3.2 ± 0.2	two hybrid assay
45	**TR4**	NR2C2	GAL4-TR4	1.76 ± 0.12	one-hybrid assay
			RIP140-VP16	2.9 ± 0.06	two hybrid assay
46	**THRα**	NR1A1	GAL4-THRa	0.97 ± 0.13	one-hybrid assay
			Thyroid hormone T-3	179.14 ± 7.84	agonist
47	**THRβ**	NR1A2	GAL4-THRb	0.19 ± 0.02	one-hybrid assay
			Thyroid hormone T-3	218.58 ± 5.06	agonist
48	**VDR**	NR1I1	GAL4-VDR	0.78 ± 0.03	one-hybrid assay
			1,25-dihydroxyvitamin D3	606.99 ± 22.31	agonist

Table S1 cont'd

Table S1. The basal activity and responsiveness of GAL4-NR vectors. The individual constructs were assessed in the SEAP reporter gene assay. The GAL4-NR vectors were cotransfected with the GAL4 SEAP reporter plasmid and the SEAP activity was assessed in cell growth medium. The data show the activity of GAL4-NR vs. that of the GAL4 expression vector in unstimulated cells or in the presence of saturating concentrations of indicated NR agonists or antagonists. For NRs with unknown ligands, we used a two-hybrid assay, co-transfecting the GAL4-NR vectors with indicated corepressor (SMRT-VP16, NCOR-VP16) or coactivator (RIP140-VP16) plasmids. The maximal fold change activity values are shown. The data are average values+/- SD of at least three independent assays.

The two-hybrid SEAP assay

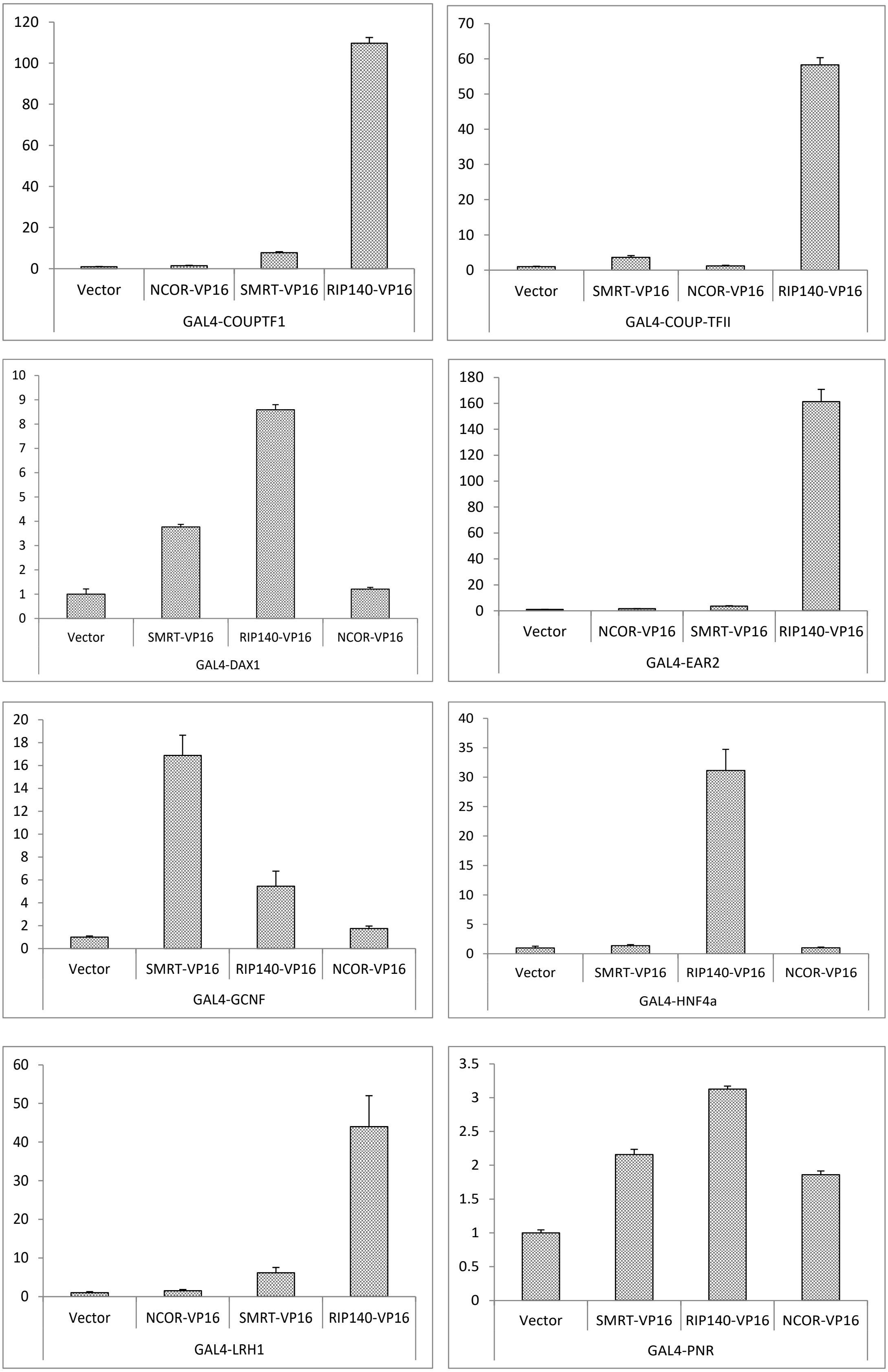

Figure S1

The two-hybrid SEAP assay

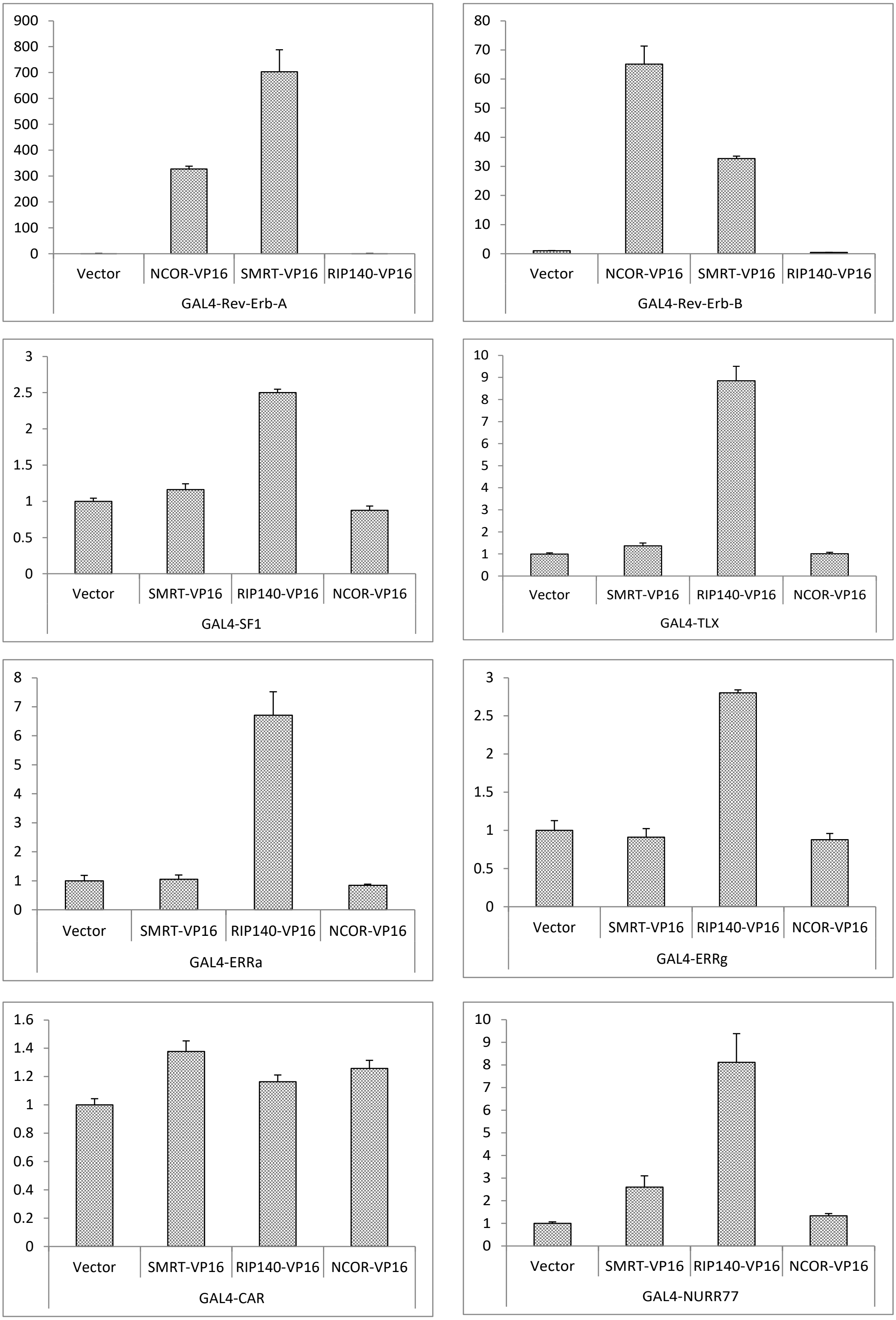

Figure S1 cont'd.

The two-hybrid SEAP assay

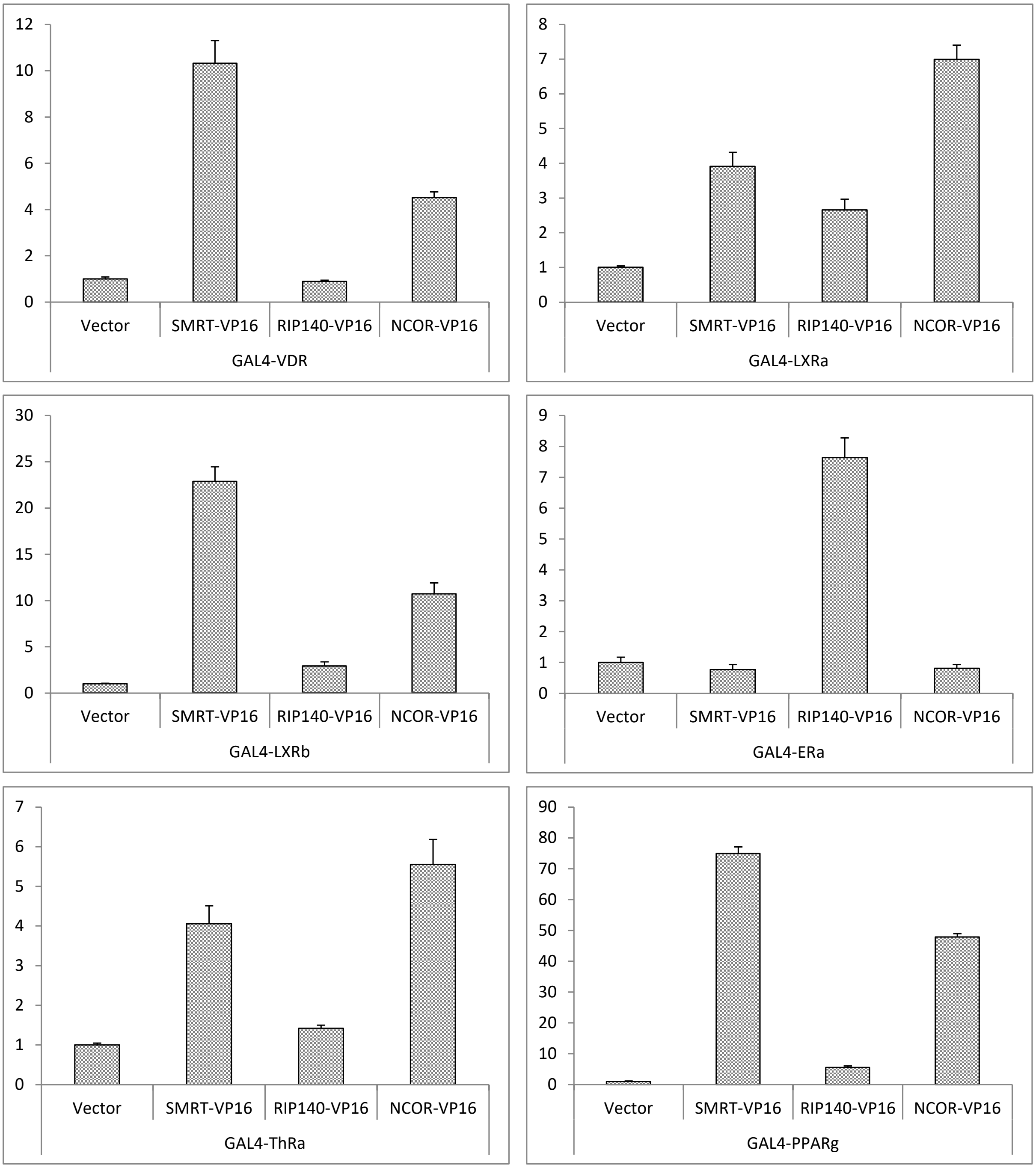

Figure S1. Assessing the functionality of the FACTORIAL NR GAL4-NR proteins by two-hybrid SEAP reporter gene assay. The GAL4-SEAP reporter was co-transfected with expression vectors for tested GAL4-NRs and the indicated NR co-factors (SMRT-VP16, NCOR-VP16, RIP140-VP16, or the VP16 (*vector*)). The SEAP induction was normalized to that in VP16-expressing cells. Average values of three independent SEAP assays+/- SD are shown.

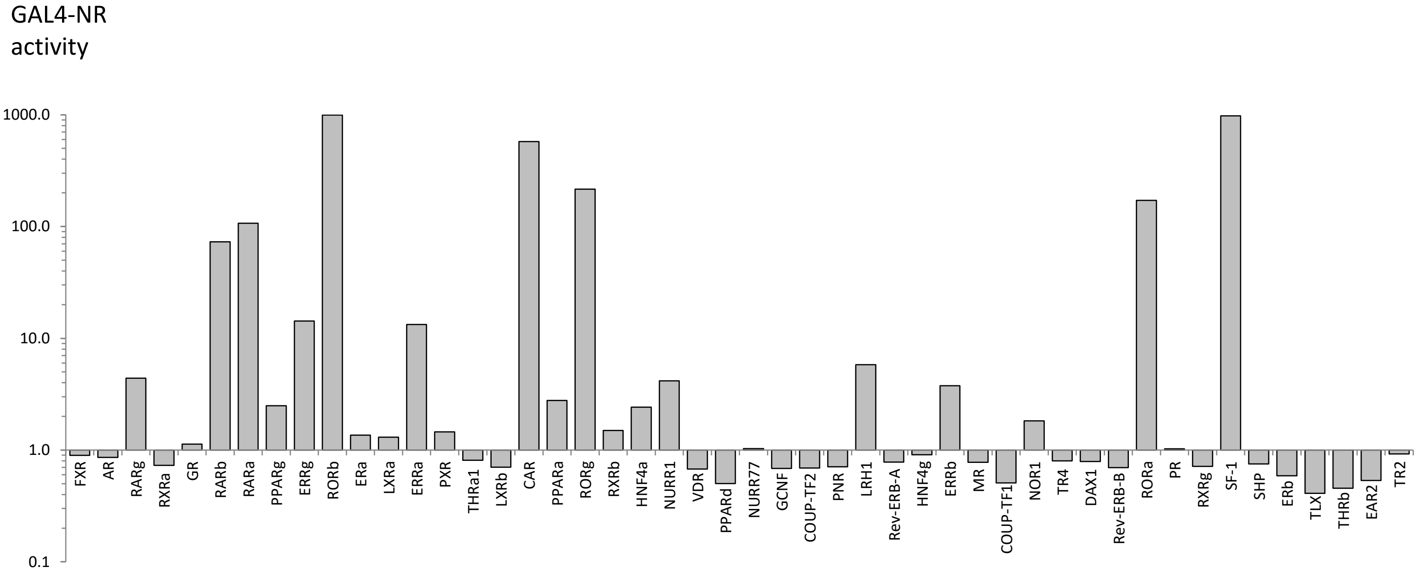

Figure S2. The basal NR activity profile of the FACTORIAL NR assay in HepG2 cells. The NR activity profile in unstimulated HepG2 cells was obtained according to FACTORIAL detection protocol (see Methods). The profile was calculated by dividing the activity of GAL4-NR modules by that of the GAL4 module. The NR activities are shown on a log scale. An average profile of three independent FACTORIAL NR assays is shown.

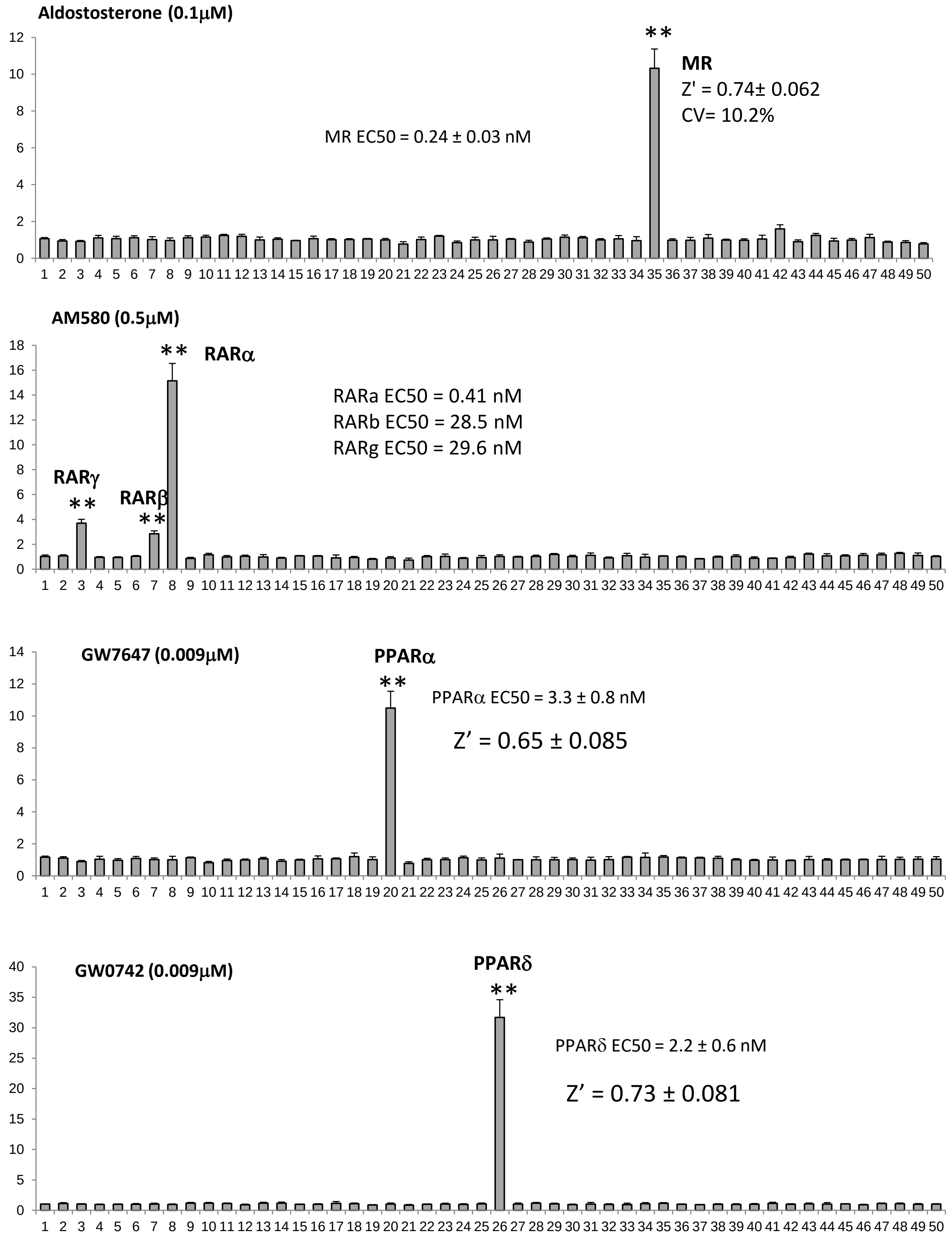

#	NR LBD
1	FXR
2	AR
3	RARγ
4	GAL4
5	RXRα
6	GR
7	RARβ
8	RARα
9	PPARγ
10	ERRα
11	RORβ
12	ERα
13	LXRα
14	ERRα
15	TATA
16	PXR
17	THRα
18	LXRβ
19	CAR
20	PPARα
21	RORα
22	RXRβ
23	HNF4α
24	NURR1
25	VDR
26	PPARδ
27	NUR77
28	GCNF
29	COUP-TFII
30	PNR
31	LRH1
32	Rev-Erbα
33	HNF4
34	ERRβ
35	MR
36	COUP-TFI
37	NOR1
38	TR4
39	DAX1
40	Rev-Erbβ
41	RORα
42	PR
43	RXRγ
44	SF-1
45	SHP
46	ERβ
47	TLX
48	THRβ
49	EAR2
50	TR2

Fig. S3. The NR activity profiles of physiological and synthetic NR ligands. The NR activity profiles were obtained as described by figure legends of Fig. 1. Significant NR responses are marked (**P<0.01; *P<0.05).

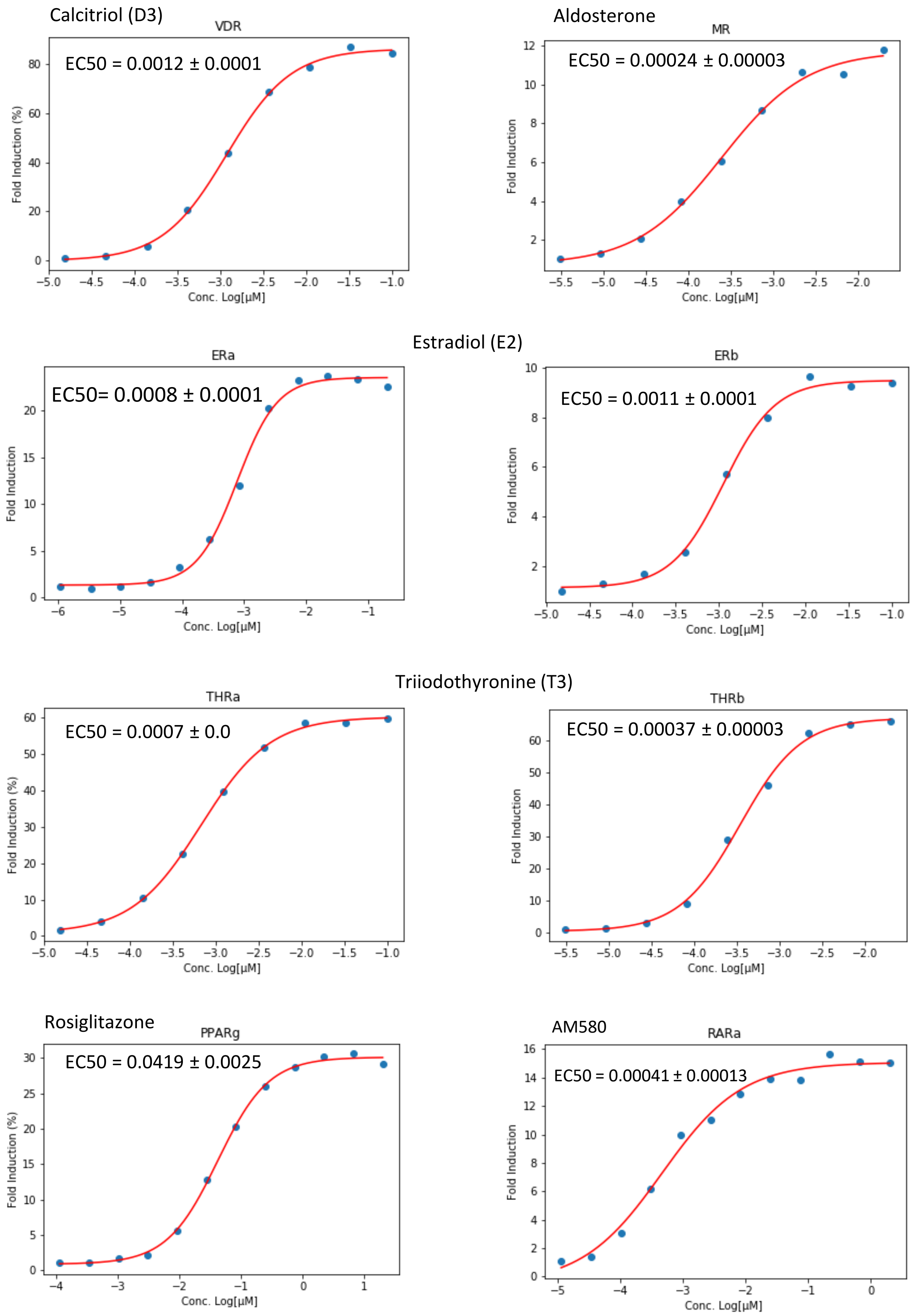

Calcitriol (D3)
VDR
EC50 = 0.0012 ± 0.0001
Fold Induction (%)
Conc. Log[μM]
Aldosterone
MR
EC50 = 0.00024 ± 0.00003
Fold Induction
Conc. Log[μM]
Estradiol (E2)
ERa
EC50= 0.0008 ± 0.0001
Fold Induction
Conc. Log[μM]
ERb
EC50 = 0.0011 ± 0.0001
Fold Induction
Conc. Log[μM]
Triiodothyronine (T3)
THRa
EC50 = 0.0007 ± 0.0
Fold Induction (%)
Conc. Log[μM]
THRb
EC50 = 0.00037 ± 0.00003
Fold Induction
Conc. Log[μM]
Rosiglitazone
PPARg
EC50 = 0.0419 ± 0.0025
Fold Induction (%)
Conc. Log[μM]
AM580
RARa
EC50 = 0.00041 ± 0.00013
Fold Induction
Conc. Log[μM]

Figure S4

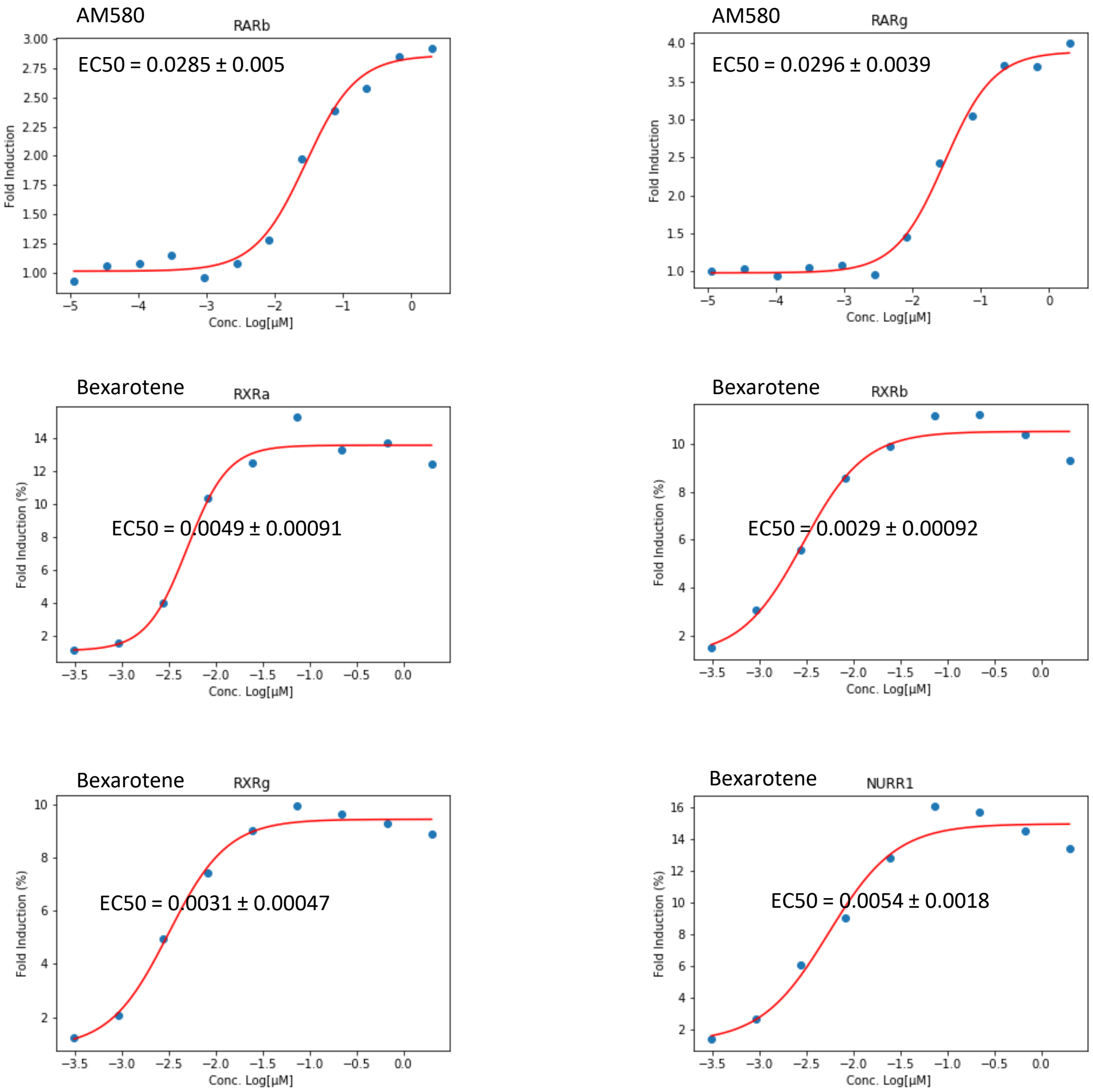

Fig. S4. Assessing the EC50 values by the FACTORIAL NR assay. Concentration-responses of FACTORIAL NR endpoints were assessed as described by the figure legend of Fig. 3. Average data of three independent FACTORIAL NR assays are shown.

A

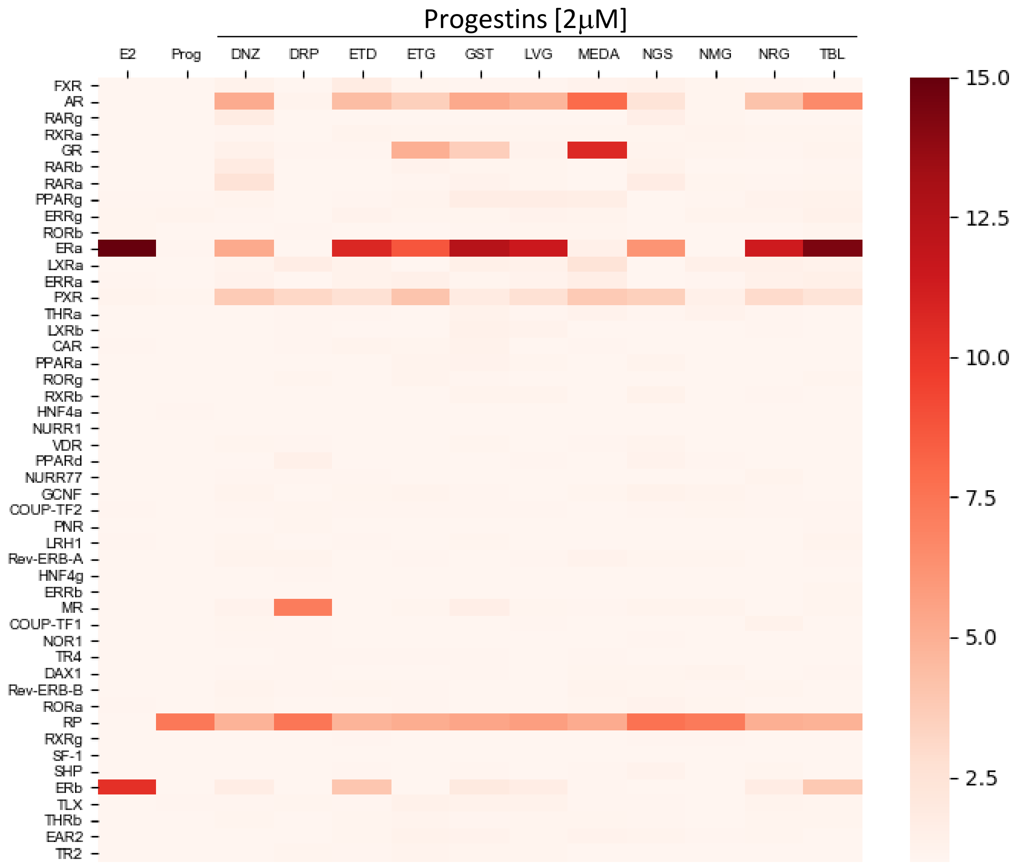

B

Relative Emax vs. hormones

	AR	ERα	MR	PR	ERβ
**DNZ**	65.31	54.81		68.33	19.06
**DRP**	19.43		80.44	132.80	
**ETD**	42.13	74.65	13.67	124.03	73.61
**ETG**	55.41	56.68		94.26	14.70
**GST**	79.92	84.22		129.75	40.34
**LVG**	60.71	77.75	16.01	134.60	31.16
**MEDA**	97.24	10.03		95.82	23.82
**NGS**	29.07	66.39		104.44	
**NMG**				118.81	
**NRG**	54.57	67.53	13.11	118.41	35.11
**TBL**	75.77	99.99		107.61	78.93

Legend:
E2 - Estradiol
Prog - Progesterone
DNZ - Danazol
DRP - Drospirenone
ETD - Ethynodiol diacetate
ETG - Etonogestrel
GST - Gestodene
LVG - Levonorgestrel
MEDA - Medroxyprogesterone Acetate
NGS - Norgestimate
NMG - Nomegestrol
NRG - Norgestrel
TBL - Tibolone

Figure S5A,B

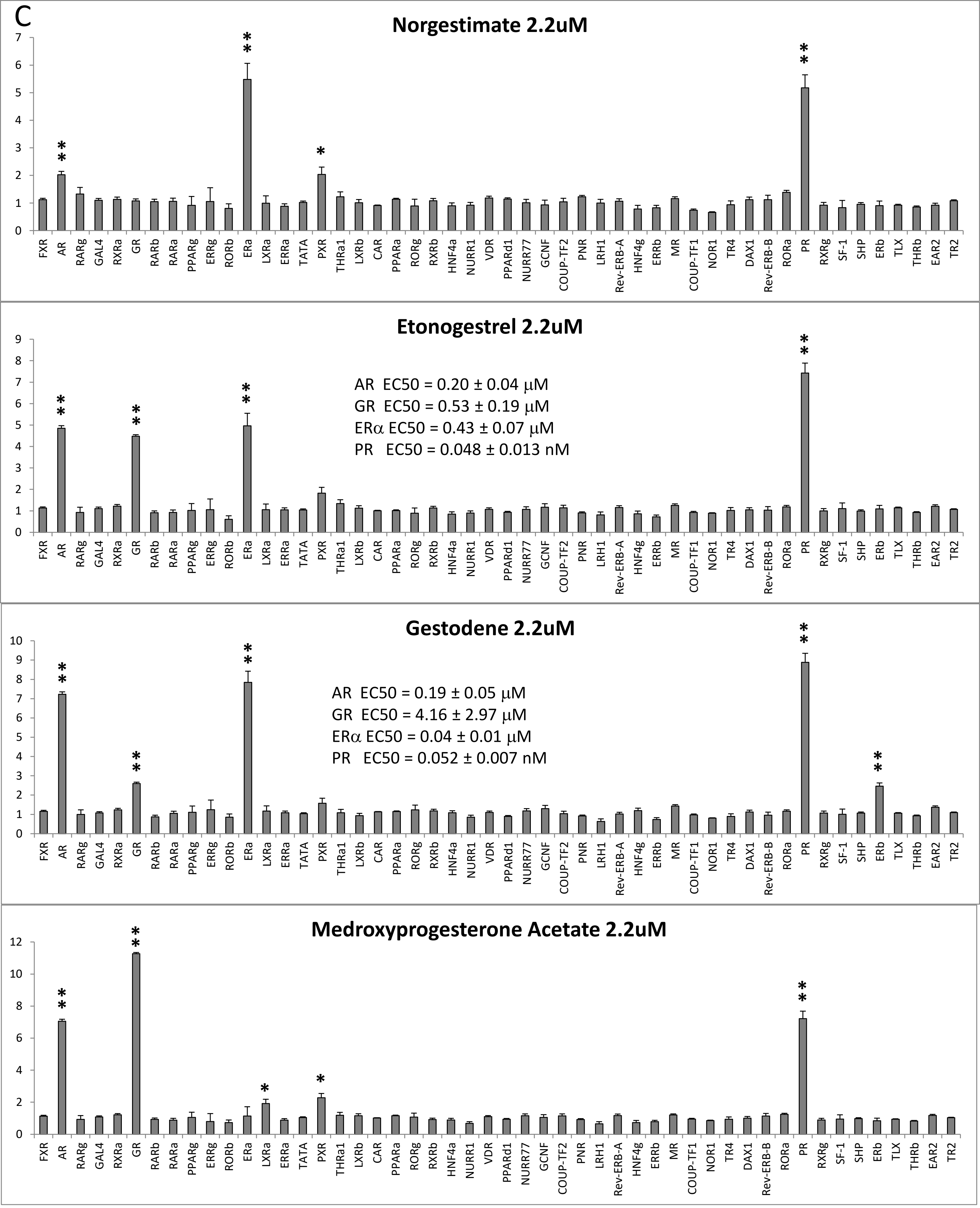
C
Norgestimate 2.2uM
Etonogestrel 2.2uM
AR EC50 = 0.20 ± 0.04 μM
GR EC50 = 0.53 ± 0.19 μM
ERα EC50 = 0.43 ± 0.07 μM
PR EC50 = 0.048 ± 0.013 nM
Gestodene 2.2uM
AR EC50 = 0.19 ± 0.05 μM
GR EC50 = 4.16 ± 2.97 μM
ERα EC50 = 0.04 ± 0.01 μM
PR EC50 = 0.052 ± 0.007 nM
Medroxyprogesterone Acetate 2.2uM
FXR AR RARg GAL4 RXRa GR RARb RARa PPARg ERRg RORb ERa LXRa ERRa TATA PXR THRa1 LXRb CAR PPARa RORg RXRb HNF4a NURR1 VDR PPARd1 NURR77 GCNF COUP-TF2 PNR LRH1 Rev-ERB-A HNF4g ERRb MR COUP-TF1 NOR1 TR4 DAX1 Rev-ERB-B RORa PR RXRg SF-1 SHP ERb TLX THRb EAR2 TR2

Figure S5C

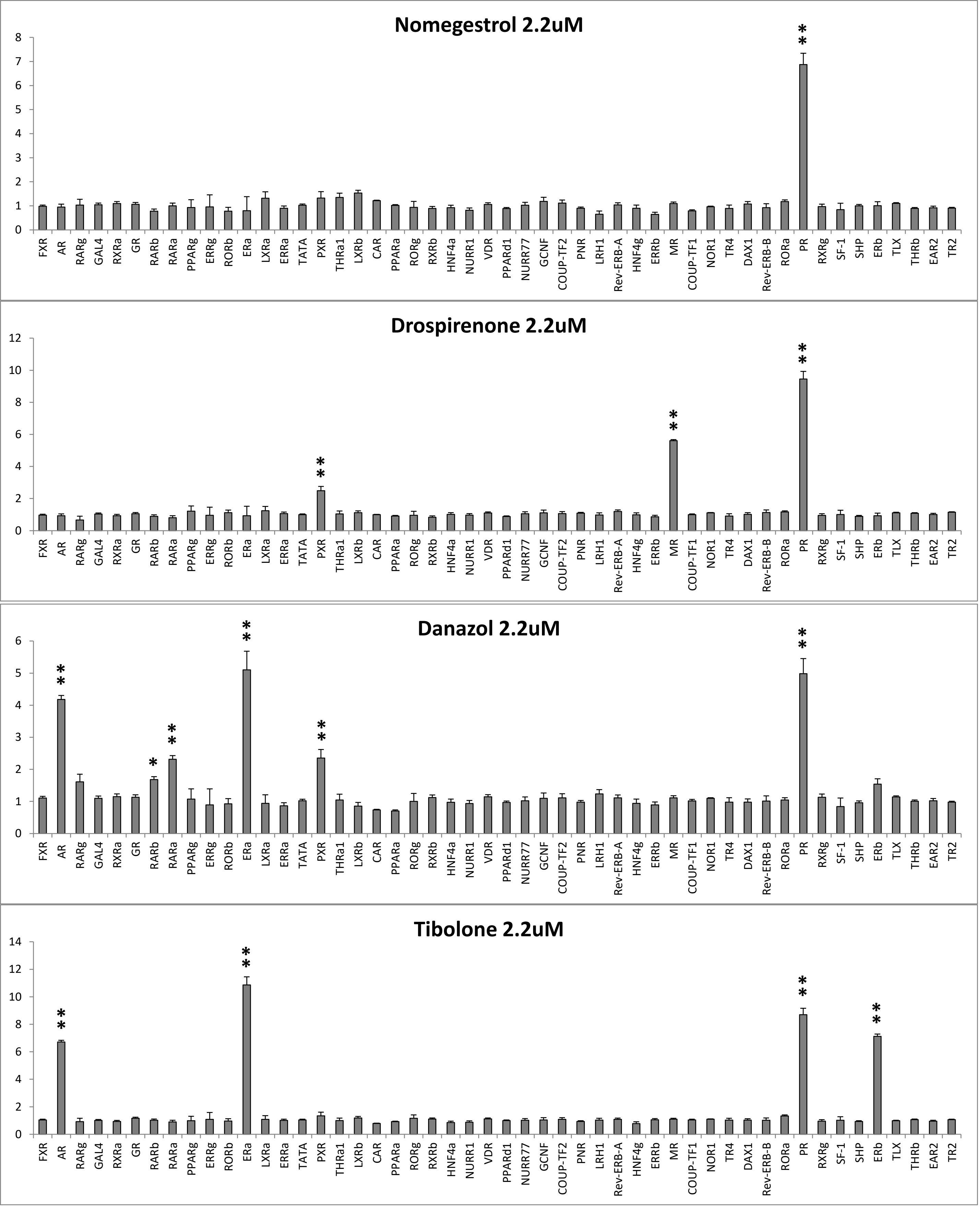

Figure S5C cont'd

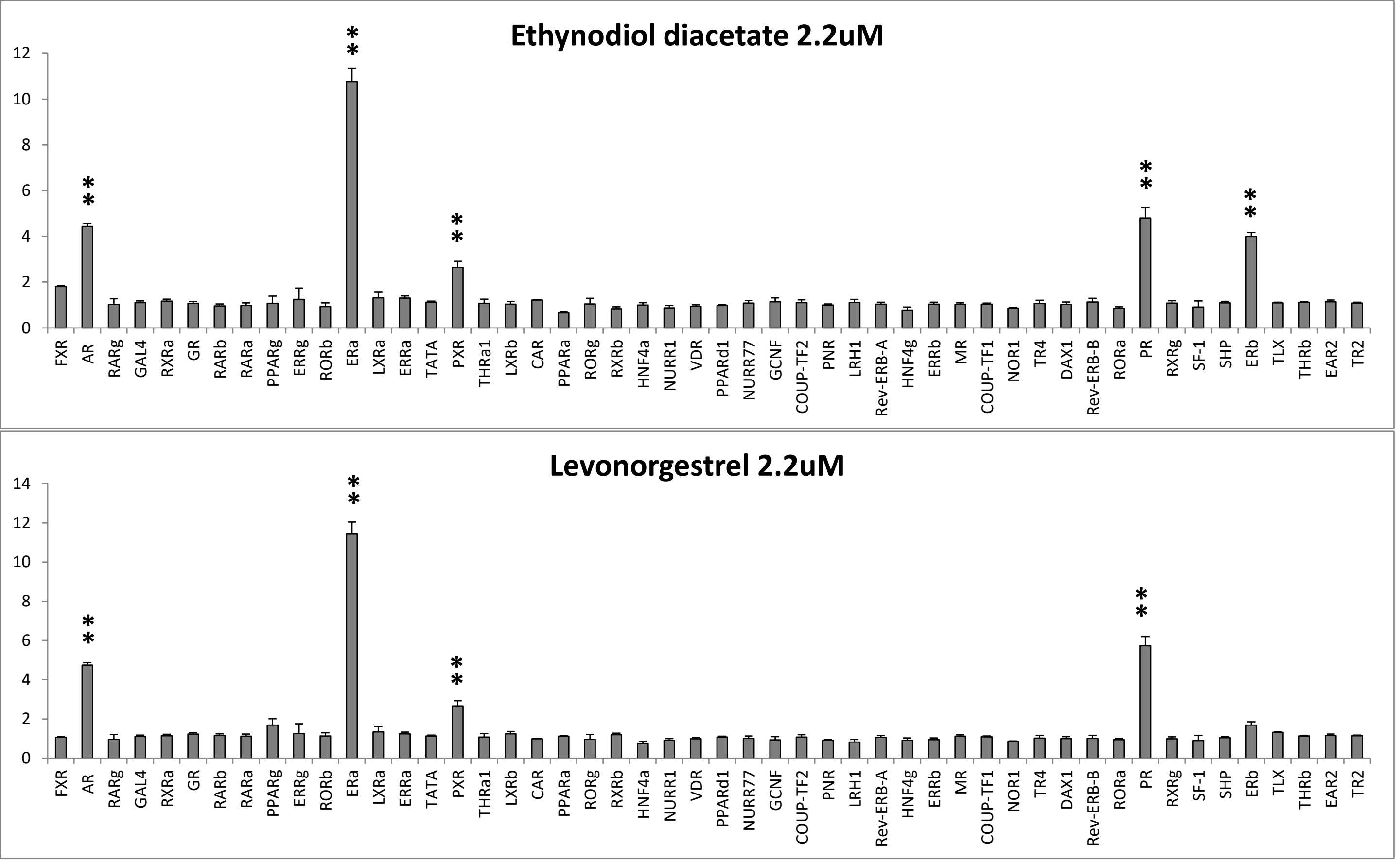

Fig. S5. The NR activity profiles of progestins. The NR activity profiles for progestins show the GAL4-NR activity in stimulated cells normalized to that in vehicle-treated cells. **A.** The heatmap shows NR responses to progestins at 2.2μM (see figure legend for Fig. 5). **B.** Table summarizes relative Emax activity of synthetic progestins at 20μM (or lower) as percentage of corresponding native hormones (testosterone (AR), estradiol (ERα, ERβ), progesterone (PR) and aldosterone (MR)). **C.** The NR activity profiles are shown as the bar graphs at 2.2μM concentration with the error bars showing standard deviation. The profiles are average data of three independent FACTORIAL NR assays. Significant NR responses are marked (**P<0.01; *P<0.05).

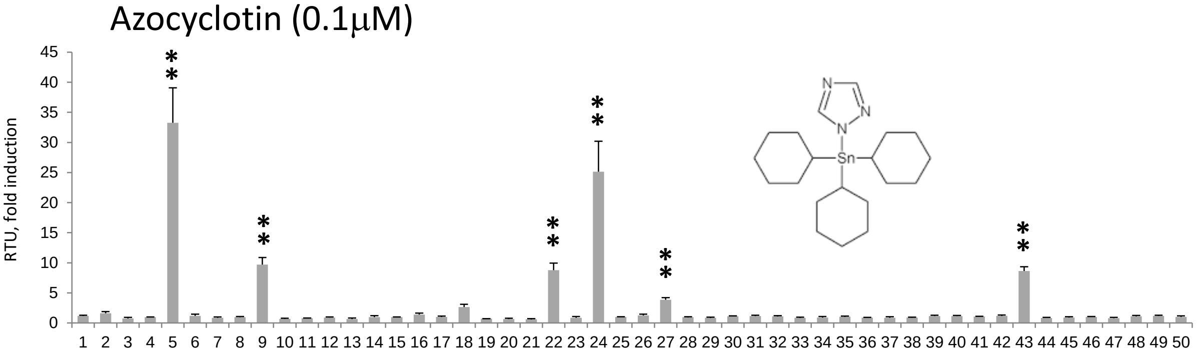

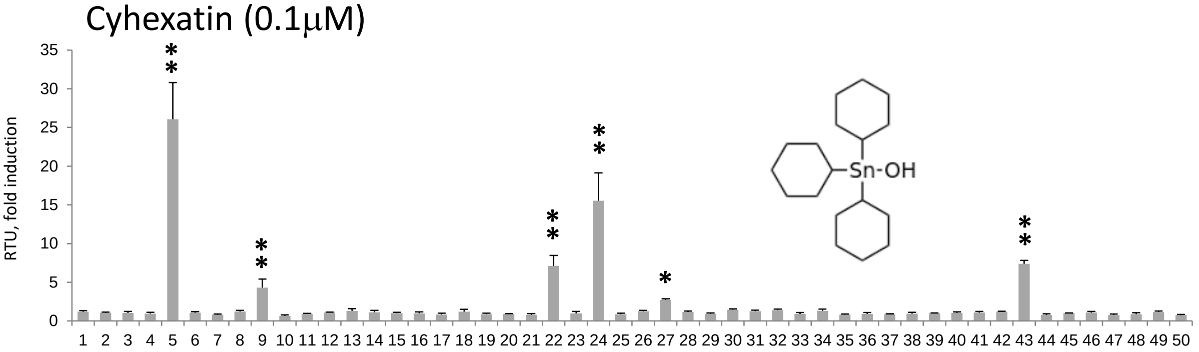

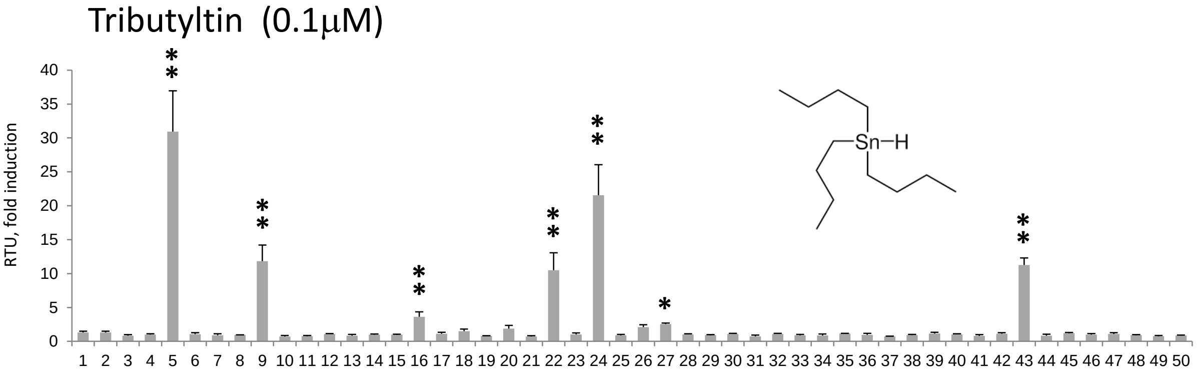

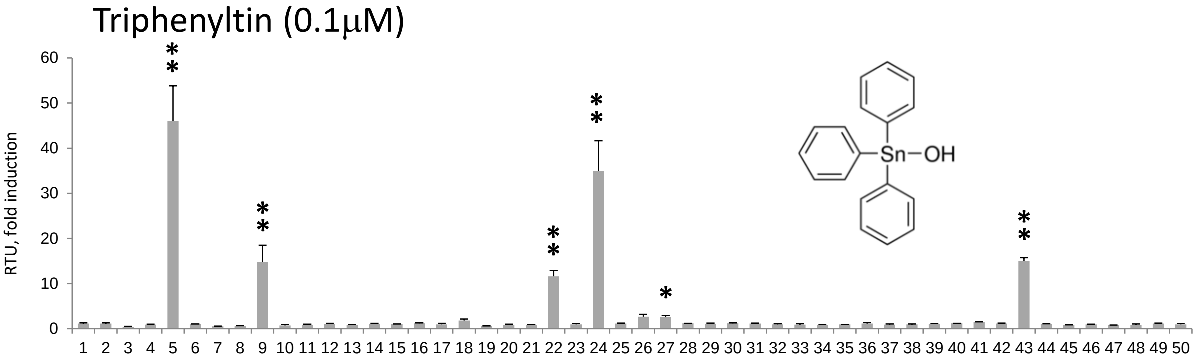

#	Name
1	FXR
2	AR
3	RARγ
4	GAL4
5	RXRα
6	GR
7	RARβ
8	RARα
9	PPARγ
10	ERRγ
11	RORβ
12	ERα
13	LXRα
14	ERRα
15	TATA
16	PXR
17	THRα1
18	LXRβ
19	CAR
20	PPARα
21	RORγ
22	RXRβ
23	HNF4α
24	NURR1
25	VDR
26	PPARδ1
27	NURR77
28	GCNF
29	COUP-TF2
30	PNR
31	LRH1
32	Rev-ERB-A
33	HNF4γ
34	ERRβ
35	MR
36	COUP-TF1
37	NOR1
38	TR4
39	DAX1
40	Rev-ERB-B
41	RORα
42	PR
43	RXRγ
44	SF-1
45	SHP
46	ERβ
47	TLX
48	THRβ
49	EAR2
50	TR2

Fig. S6. The NR activity profiles of organotins. The NR activity profiles show NR responses in stimulated cells normalized to those in vehicle-treated cells. The bar graphs show average data of three independent FACTORIAL NR assays+/- SD values. Significant NR responses are marked (**P<0.01; *P<0.05).

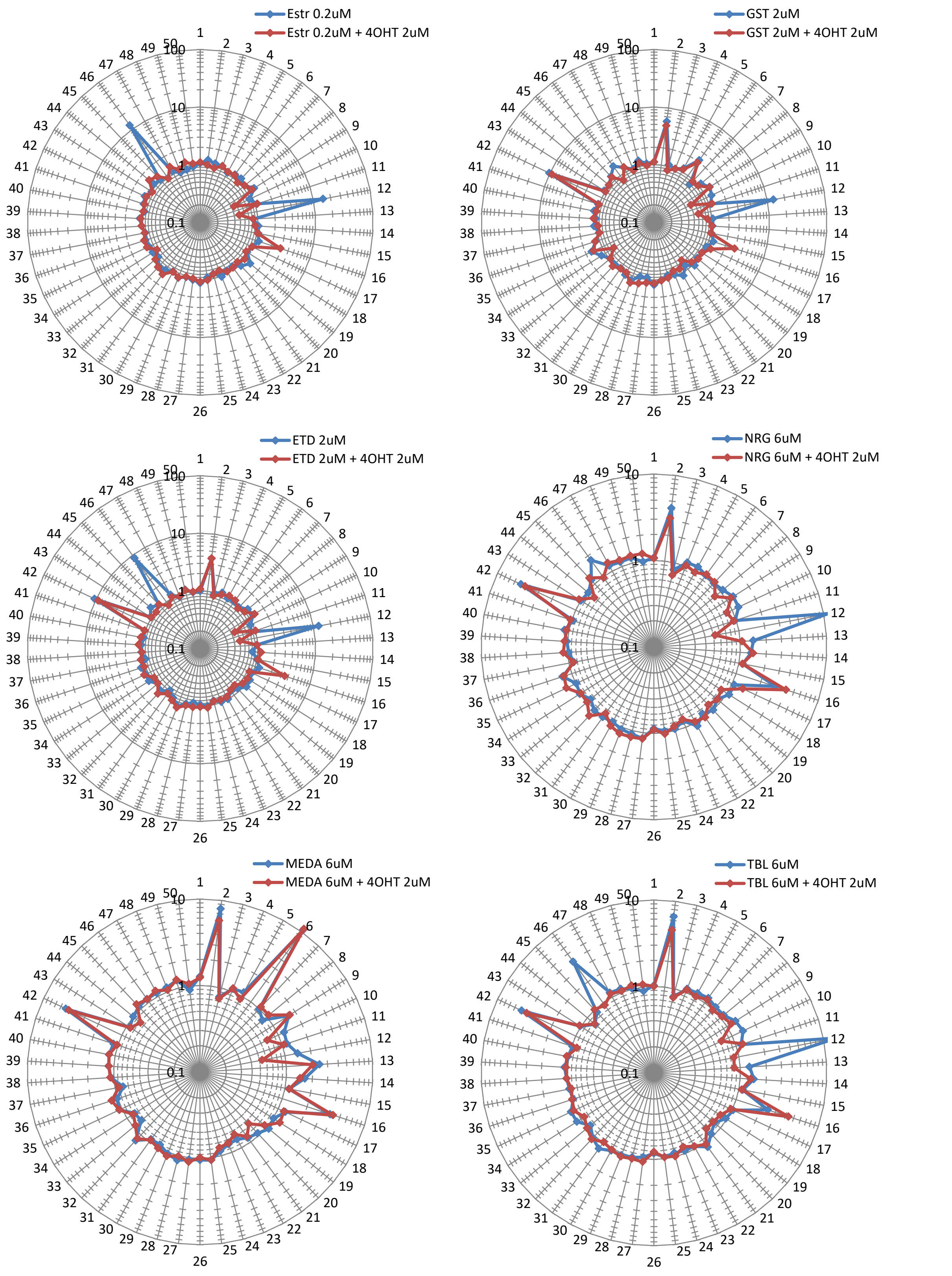

#	Name
1	FXR
2	AR
3	RARγ
4	GAL4
5	RXRα
6	GR
7	RARβ
8	RARα
9	PPARγ
10	ERRγ
11	RORβ
12	ERα
13	LXRα
14	ERRα
15	TATA
16	PXR
17	THRα1
18	LXRβ
19	CAR
20	PPARα
21	RORγ
22	RXRβ
23	HNF4α
24	NURR1
25	VDR
26	PPARδ1
27	NURR77
28	GCNF
29	COUP-TF2
30	PNR
31	LRH1
32	Rev-ERB-A
33	HNF4γ
34	ERRβ
35	MR
36	COUP-TF1
37	NOR1
38	TR4
39	DAX1
40	Rev-ERB-B
41	RORα
42	PR
43	RXRγ
44	SF-1
45	SHP
46	ERβ
47	TLX
48	THRβ
49	EAR2
50	TR2

Fig. S7. Examining mechanisms for the off-target progestin activity. The NR activity profiles were obtained using the FACTORIAL NR in a competitive mode, as described by figure legend for Fig. 7C. The blue line graphs show NR activity fold-changes in progestin- vs. vehicle-treated cells after a 24-h treatment. The red line graphs show the NR responses to progestins in the presence of ER inhibitor 4-Hyrdoxytamoxifen (4-HT). Average profiles of three independent replicate FACTORIAL NR assays are shown.

A

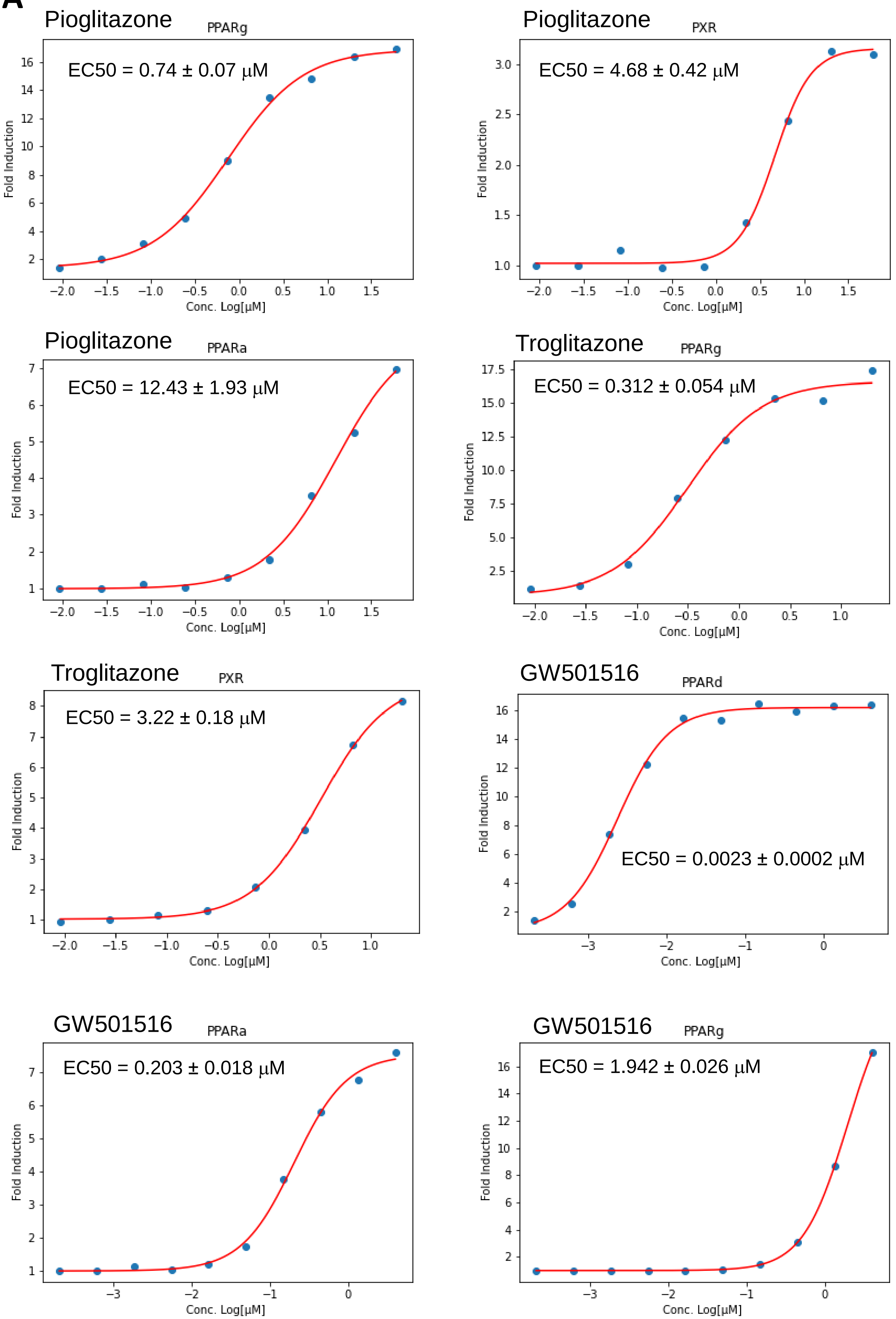
Pioglitazone PPARg
EC50 = 0.74 ± 0.07 µM
Pioglitazone PXR
EC50 = 4.68 ± 0.42 µM
Pioglitazone PPARa
EC50 = 12.43 ± 1.93 µM
Troglitazone PPARg
EC50 = 0.312 ± 0.054 µM
Troglitazone PXR
EC50 = 3.22 ± 0.18 µM
GW501516 PPARd
EC50 = 0.0023 ± 0.0002 µM
GW501516 PPARa
EC50 = 0.203 ± 0.018 µM
GW501516 PPARg
EC50 = 1.942 ± 0.026 µM
Fold Induction
Conc. Log[µM]

Fig. S8A

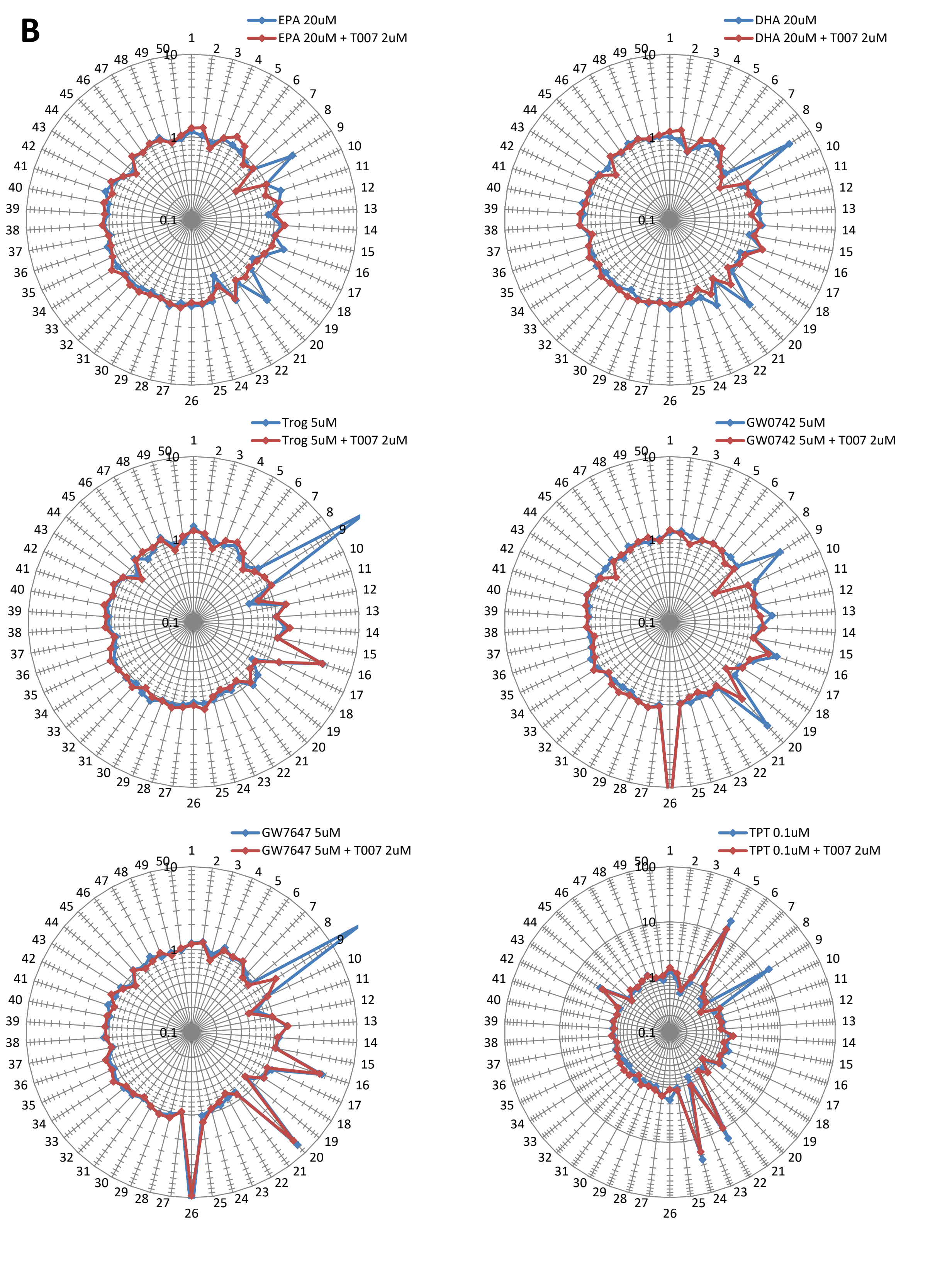

#	Name
1	FXR
2	AR
3	RARγ
4	GAL4
5	RXRα
6	GR
7	RARβ
8	RARα
9	PPARγ
10	ERRγ
11	RORβ
12	ERα
13	LXRα
14	ERRα
15	TATA
16	PXR
17	THRα1
18	LXRβ
19	CAR
20	PPARα
21	RORγ
22	RXRβ
23	HNF4α
24	NURR1
25	VDR
26	PPARδ1
27	NURR77
28	GCNF
29	COUP-TF2
30	PNR
31	LRH1
32	Rev-ERB-A
33	HNF4γ
34	ERRβ
35	MR
36	COUP-TF1
37	NOR1
38	TR4
39	DAX1
40	Rev-ERB-B
41	RORα
42	PR
43	RXRγ
44	SF-1
45	SHP
46	ERβ
47	TLX
48	THRβ
49	EAR2
50	TR2

Fig. S8. Examining mechanisms for the off-target activity of PPAR agonists. The NR activity profiles were obtained after a 24 h incubation with indicated concentrations of PPAR ligands. **A.** Concentration-response curves of on-target and off-target effects of PPAR agonists. **B**. The blue line graphs show NR activity fold changes in the stimulated vs. vehicle-treated cells. The red line graphs show NR responses to the PPAR ligands in the presence of a PPAR inhibitor T0070907 (T007). Average profiles of three independent replicate FACTORIAL NR assays are shown. *Abbreviations: EPA,* eicosapentaenoic acid; *DHA,* docosahexaenoic acid; *Trog,* troglitazone; *TPT,* triphenyltin.
